# Molecular bases of responses to abiotic stress in trees

**DOI:** 10.1093/jxb/erz532

**Published:** 2019-11-26

**Authors:** Maximiliano Estravis-Barcala, María Gabriela Mattera, Carolina Soliani, Nicolás Bellora, Lars Opgenoorth, Katrin Heer, María Verónica Arana

**Affiliations:** 1 Instituto Andino Patagónico de Tecnologías Biológicas y Geoambientales, (Consejo Nacional de Investigaciones Científicas y Técnicas- Universidad Nacional del Comahue), San Carlos de Bariloche, Rio Negro, Argentina; 2 Instituto de Investigaciones Forestales y Agropecuarias Bariloche (Instituto Nacional de Tecnología Agropecuaria - Consejo Nacional de Investigaciones Científicas y Técnicas), San Carlos de Bariloche, Rio Negro, Argentina; 3 Department of Ecology, Philipps University Marburg, Marburg, Germany; 4 Swiss Federal Research Institute WSL, Birmensdorf Switzerland; 5 Department of Conservation Biology, Philipps University Marburg, Marburg Germany; 6 Pontificia Universidad Catolica de Chile, Chile

**Keywords:** Abiotic stress, drought, epigenomics, global climate change, population genomics, temperature, transcriptomics, trees

## Abstract

Trees are constantly exposed to climate fluctuations, which vary with both time and geographic location. Environmental changes that are outside of the physiological favorable range usually negatively affect plant performance and trigger responses to abiotic stress. Long-living trees in particular have evolved a wide spectrum of molecular mechanisms to coordinate growth and development under stressful conditions, thus minimizing fitness costs. The ongoing development of techniques directed at quantifying abiotic stress has significantly increased our knowledge of physiological responses in woody plants. However, it is only within recent years that advances in next-generation sequencing and biochemical approaches have enabled us to begin to understand the complexity of the molecular systems that underlie these responses. Here, we review recent progress in our understanding of the molecular bases of drought and temperature stresses in trees, with a focus on functional, transcriptomic, epigenetic, and population genomic studies. In addition, we highlight topics that will contribute to progress in our understanding of the plastic and adaptive responses of woody plants to drought and temperature in a context of global climate change.

## Introduction

Forests play a crucial role for the climate at various temporal-spatial scales. For example, forests directly affect the local climate by interacting with biogeochemical water cycles (Ellison *et al.*, 2017) whilst at the same time influencing the global carbon cycle because they hold a large fraction of the global carbon stock, acting as a major sink for atmospheric CO_2_ (Pan *et al.*, 2011). As a result of climate change, forest biomes are expected to face increasing temperatures, extreme cold winters with harsh springs, and/or more frequent and severe droughts (Sheffield and Wood, 2007; Loarie *et al.*, 2009; Li *et al.*, 2015). These extreme abiotic conditions are expected to have significant consequences for biodiversity, primary productivity, and ecological functions (Ciais *et al.*, 2005; Sitch *et al.*, 2008). Most of our knowledge about the molecular bases of the responses of plants to abiotic stresses comes from studies of annual plants such as Arabidopsis and crop species, and information about trees is generally scarce and mostly limited to model genera such as *Populus* (Harfouche *et al.*, 2014). An important question therefore remains as to how trees will be able to cope with the extreme environmental conditions that are expected to be aggravated under global climate change.

Our knowledge of the physiological responses of woody plants to abiotic stresses has significantly increased with the ongoing development of measurement techniques, for example the Scholander pressure bomb, which was first used for measuring drought stress in a tree more than 50 years ago (Waring and Cleary, 1967). The increasing availability of the genomes of tree species (Sow *et al.*, 2018, and see https://plabipd.de for updates) in combination with advances in next generation sequencing (NGS) techniques, which allow in-depth molecular studies of non-model species by making it straightforward to obtain massive quantities of DNA and RNA sequence data from virtually any tissue and developmental and/or physiological conditions (Metzker, 2010), provide a powerful tool for advancing our knowledge of the genetic architecture that underlies abiotic stress responses in trees. In this review, we describe and integrate the latest knowledge on drought and temperature stress in trees, since they are proposed to be the major factors that will have an impact according to global climate predictions. We focus on studies based on transcriptomics, epigenomics, and population genomics that have mostly been carried out over the last decade, with the aim of discussing molecular mechanisms and the way in which they may contribute to tree responses to stress in a context of global climate change.

## Concepts and main features related to the physiology of plant stress

Plant stress can be defined as any unfavorable condition or substance that affects or blocks the metabolism, growth, or development of the plant (Lichtenthaler, 1996). However, from a functional point of view, many physiologists define stress as the altered physiological condition caused by factors that tend to change an equilibrium (reviewed by Kranner *et al.*, 2010). In this review, we distinguish external stress factors from internal stresses whenever possible, and refer to abiotic stress as the environmental condition that triggers changes in the physiology of the organism, whereas the physiological conditions caused by stressing factors are referred to as the response to stress. The particular stress caused by temperature is referred to as thermal or temperature stress, whilst the stress caused by drought is termed drought stress.

The physiological response to stress may vary depending on the frequency and intensity of the stressful condition and the ontological stage of the plant. Importantly, not only do different tree species vary considerably in their inherent ability to resist stress, but the resistance of individual plants also changes dramatically during the year, for example as reported extensively for cold stress (Weiser, 1970; Harrison *et al.*, 1978). This raises the concept that stress responses in trees may show temporal variations, which can be regulated diurnally (Hüve *et al.*, 2006) or seasonally (Harrison *et al.*, 1978). When the exposure of the plant to a gradually increasing stressful environment induces physiological adjustment that protects it from the growth inhibition and/or injury that occur when environmental stresses are abruptly imposed, but does not involve changes in the DNA sequence, the response is referred to as hardening or acclimation (Kranner *et al.*, 2010; Crisp *et al.*, 2016). When hardening persists between generations, the process is referred to as adaptive transgenerational plasticity (Herman and Sultan, 2011). In harsh environments, under constant stressful conditions, genetic changes can be fixed over many generations by selective environmental pressure, and in this case, populations show adaptation to the environment (Mitchell-Olds *et al.*, 2007). Acclimation, adaptive transgenerational plasticity, and genetic adaptation can contribute simultaneously to the overall tolerance of the plant to extremes in the abiotic environment.

### Drought stress

Drought stress is an important driver of tree mortality (Park Williams *et al.*, 2013; McDowell *et al.*, 2018) and trees have evolved a variety of strategies to respond to and cope with the different stages of water deficit, which involve biochemical, physiological, and morphological adjustments. These can be classified into drought avoidance and drought tolerance. Drought avoidance is mainly based on strategies directed at avoiding low water potentials, which involve mechanisms that maintain the water status of the plant by ensuring continued water uptake and minimizing water loss. These involve constitutive responses, such as the expression of barriers that decreases water evaporation (Aharoni *et al.*, 2004), and inducible responses including turgor-dependent decreases in cell division and expansion, which reduce leaf area (Marron *et al.*, 2003), and stomatal regulation, which reduces transpiration, albeit to the detriment of CO_2_ uptake (Choat *et al.*, 2018). When drought avoidance mechanisms are not enough to mitigate the stress, plants respond by activating drought tolerance mechanisms that are directed to protecting tissues against damage, mainly through the induction of components that protect cellular elements against dehydration, mechanisms that promote osmotic adjustment, and detoxification of reactive oxygen species (ROS) (Du *et al.*, 2009; Claeys and Inzé, 2013). It is important to note that although several general principles related to drought stress are valid for both gymnosperms and angiosperms, gymnosperms are generally considered to be more drought resistant than angiosperms, and this is linked to differences in traits such as xylem structure and stomatal regulation. On the other hand, angiosperms show more complex anatomical responses to drought than gymnosperms (reviewed by Moran *et al.*, 2017)

### Temperature stress

Temperature is one of the major environmental factors that constrains the geographical distribution of organisms, and it is in large part governed by latitude and altitudinal gradients, which determine thermal niches with specific characteristics. Stress as a result of both low and high temperatures has a direct impact on molecular (DNA, lipid, proteins) and macro-molecular (membranes, chromosomes) structures, principally due to a thermodynamic effect (Ruelland and Zachowski, 2010).

#### Cold stress

Low temperature is one of the main factors that limits the productivity and geographical distribution of many species, including important agricultural crops. The ability of trees to grow at low temperatures can be related to their capacity to cope with sub-zero temperatures through mechanisms that promote frost resistance, or with relatively cold temperatures in a range between 0 °C and ~15° through processes that trigger chilling resistance. Whereas frost response mechanisms are frequently present in tree species that belong to temperate and boreal ecosystems, trees from tropical environments express chilling symptoms in a range between 0 °C and 10–15 °C (Graham and Patterson, 1982; Allen *et al.*, 2000; Mai *et al.*, 2010). Chilling and freezing tolerance are related to the development of mechanisms that impair low-temperature damage, such as modification of the saturation level of the fatty-acid chains in membrane lipids (Falcone *et al.*, 2004), which allows cells to maintain membrane fluidity at low temperatures. In woody plants, different parts of the plant and even adjacent tissues show differences in cold hardiness. For example, in stems, living cells in the wood are often less resistant by several degrees in midwinter than those of bark tissues (reviewed by Weiser, 1970). In particular, bud dormancy in deciduous trees is a trait that promotes survival during harsh climatic conditions (Cooke *et al.*, 2012). Environmental factors such as short days and low temperature promote bud dormancy and cold hardiness in trees, and it has been demonstrated that the circadian clock is an essential timer that regulates seasonal growth and cold hardiness in deciduous tree species (Ibáñez *et al.*, 2010; Johansson *et al.*, 2014).

#### Heat stress

High temperatures exert an important constraint for tree growth and development, and heat stress is an important driver of tree mortality in natural ecosystems (McDowell *et al.*, 2018). Long-term adaptive strategies for heat tolerance include decreasing the leaf canopy temperature through increasing evaporative cooling by enhancing leaf number and area, through changing leaf orientation, through the development of reflective trichomes and waxes, or through leaf shedding (Teskey *et al.*, 2015). At the leaf level, the processes that are affected include photosynthesis, dark respiration, photorespiration, emissions of volatile organic compounds (VOCs), stomatal conductance, and transpiration (Teskey *et al.*, 2015). At the cellular and subcellular level, the reduction in water content caused by heat has negative effects on cell division and growth (Hasanuzzaman *et al.*, 2013), on the functioning of photosystem II, the fluidity of the thylakoid membrane, and on rubisco activity (Teskey *et al.*, 2015). Injurious effects of high temperature include protein denaturation and aggregation, inactivation of enzymes, membrane dysfunction, and de-organization of microtubules, which can eventually lead to the breakdown of cell integrity.

While some of the physiological responses described above have been well characterized, much remains to be determined about their detailed mechanisms, their plasticity, and their underlying molecular regulatory networks in trees.

## Functional studies on sensing stress and downstream signaling pathways

**Fig. 1.**
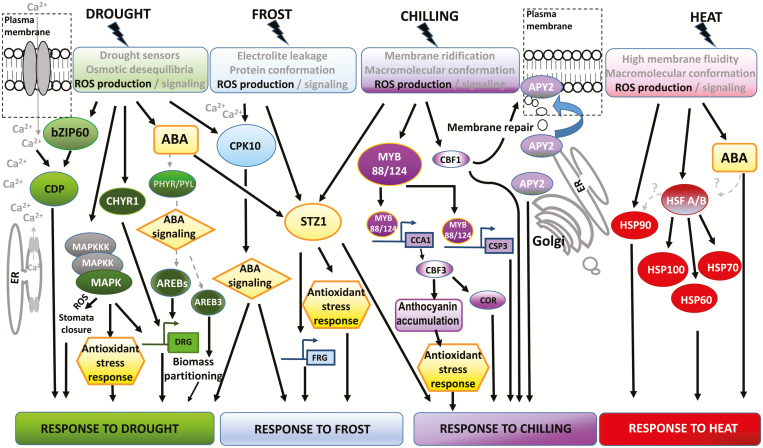
Schematic representation of the signaling transduction pathways that respond during drought and temperature stresses based on the information available for different tree species. Different colors indicate different transduction pathways: green, drought; light blue, frost; violet, chilling; red, heat. Common elements among different signaling pathways are indicated with yellow. Dashed lines and gray colouring indicate hypotheses. CDP, calcium-dependent protein kinase; COR, cold-responsive genes; DRG, drought-responsive genes; ER, endoplasmic reticulum; FRG, frost-responsive genes; ROS, reactive oxygen species.

Plants have evolved a diverse spectrum of molecular programs aimed at promoting switches in growth and development under stressful conditions, and most of our knowledge of these comes from model species such as Arabidopsis and annual crop plants (e.g. Bjornson *et al.*, 2016). Several molecules have been proposed to sense abiotic stress (Mittler *et al.*, 2012; Srivastava *et al.*, 2013; Liu *et al.*, 2017), but their roles and relevance in the stress responses of trees are still unclear. Beyond the specific receptors involved, a widely accepted concept is that abiotic stress exerts different biological impacts at the cellular level that trigger the stress-sensing mechanism in plants. There is evidence that this may also be true for trees, and reports in both woody gymnosperms and angiosperms suggest that, similar to Arabidopsis (Saidi *et al.*, 2011), calcium-dependent signaling, mitogen-activating protein kinases (MAPKs), and heat-shock proteins (Hsps) act during abiotic stress. For instance, drought stimulates the expression of the transcription factor (TF) bZIP60 in *Pinus strobus*, which is known to promote drought tolerance and to activate several calcium-dependent protein kinases in transgenic rice (Tang and Page, 2013). Similarly, in *Populus euphratica*, the calcium-dependent protein kinase 10 (CPK10) is expressed under drought and frost and activates both drought- and frost-responsive genes to induce stress tolerance (Chen *et al.*, 2013). On the other hand, in the hybrid poplar *Populus trichocarpa* × *deltoides*, MAPK cascades are involved in the promotion of antioxidant stress responses and in *P. trichocarpa* they stimulate the expression of drought-related genes (Hamel *et al.*, 2005; Wang *et al.*, 2014). The different signal-transduction components that are so far known to act during drought and temperature stresses in trees are integrated in Fig. 1, in a hypothetical scheme based on the data available from functional studies performed in different woody angiosperms and gymnosperms. Although the role of each individual component remains to be examined across all the different taxa and tissues, the figure illustrates the current state of our knowledge of this area in trees.

Physiological and functional studies in poplar indicate that heat stress induces the expression of Hsps, such as Hsp90 (Zhang *et al.*, 2013), and several are co-expressed with Heat-Shock Factors A and B (HSFA and HSFB) (Zhang *et al.*, 2015). In *P. trichocarpa*, several Hsps such as Hsp60, Hsp70, and Hsp100 are induced during the transient expression of HSFA2, HSFA6a, and HSFB2a (Zhang *et al.*, 2015), indicating that, similar to other species, HSF proteins regulate Hsps in trees.

Changes in membrane fluidity due to heat or cold also trigger temperature responses (Vaultier *et al.*, 2006; Saidi *et al.*, 2011) and the damage to membranes and the loss of ionic and osmotic homeostasis promote the production of ROS, which activate specific signaling pathways in Arabidopsis (Mittler *et al.*, 2004). In trees, an increase in ROS activity has been reported under drought, chilling, frost, and heat stress (Lei *et al.*, 2006; Chen *et al.*, 2014; Xie *et al.*, 2018; He *et al.*, 2019), and MYB and zinc-finger TFs promote the expression of ROS detoxification enzymes, thus enhancing stress tolerance. For instance, in *P. euphratica*, the cysteine-2/histidine-2-type zinc-finger TF STZ1, which is mostly expressed in young stems, phloem, and xylem, is induced by drought, frost, and chilling stress in leaves and promotes the expression of ascorbate peroxidase 2, which scavenges ROS and enhances frost tolerance. The ectopic over-expression of STZ1 improves freezing tolerance in transgenic poplar (He *et al.*, 2019). On the other hand, under drought stress, the poplar protein CHYR1, which is a homolog of an E3 ligase that is a core component of the ubiquitination pathway and contains a CHY zinc-finger domain in Arabidopsis (Ding *et al.*, 2015), promotes stomatal closure via ROS signaling (He *et al.*, 2018). This indicates that components and signaling pathways that mediate stomatal closure in response to drought are, to some extent, conserved between Arabidopsis and poplar.

Chilling in apple (*Malus* × *domestica*) induces the overexpression of the R2R3 MYB TFs MYB88 and MYB124, which stimulate anthocyanin accumulation and ROS detoxification (Xie *et al.*, 2018). This might be mediated, at least in part, through modulation of the expression of C-repeat/DREB binding factors (CBFs), which are key TFs that are members of the APETALA2/Ethylene-Responsive Factor family (AP2/ERF) and function in chilling resistance and acclimation in other species (Shi *et al.*, 2018). Both MYB88 and MYB124 bind to the promoters of apple homologs for COLD SHOCK DOMAIN PROTEIN 3 (CSP3) and CIRCADIAN CLOCK ASSOCIATED 1 (CCA1), and act as direct regulators of their expression in transient assays in tobacco leaves. CCA1 but not CSP3 activates the expression of the apple homolog for CBF3 under cold stress, indicating that MYB88 and MYB124 act to promote both the CBF and CBF- independent pathways (Xie *et al.*, 2018). In addition to CBF3, CBF1 is cold-inducible in stems and leaf tissues of poplar, whereas CBF2 and CBF4 are mostly induced in leaves (Benedict *et al.*, 2006; Li *et al.*, 2017), indicating the existence of transcriptional diversification of CBFs across different tissues. Interestingly, CBF1 transcripts increase under during drought, chilling, frost, and heat stress and studies in *P. simonii* indicate that this factor may promote chilling tolerance through the regulation of membrane-related functions (Li *et al.*, 2017). CBF4 is also induced by drought and frost in leaves in *P. euphratica* (Tian *et al.*, 2017). This suggests that CBFs may act as nodes in the expression of different stress-signaling pathways. In trees, the way in which drought, chilling, frost, and heat stress responses may be interconnected through the action of CBF factors is still unknown.

Among the phytohormones that are involved in abiotic stress responses, the action of abscisic acid (ABA) is the best functionally characterized in trees. Different abiotic stresses stimulate the accumulation of ABA (Escandón *et al.*, 2016; Pashkovskiy *et al.*, 2019) and functional analysis indicates that the main ABA signaling components are conserved between Arabidopsis and poplar (Papacek *et al.*, 2017). In *P. tremula* × *tremuloides*, ABA signaling plays a crucial role in the trade-off of biomass allocation under drought stress, and ABA is proposed to play an important role in regulating the biomass of woody plants, mostly by affecting leaf area (Yu *et al.*, 2019). ABA induces the expression of components that participate in the responses to drought, frost, and chilling such as the STZ1 TF, as mentioned above (He *et al.*, 2019), and CPK10 promotes ABA signaling during drought and frost (Chen *et al.*, 2013; Li *et al.*, 2019). The relevance of ABA signaling in the responses of woody plants to abiotic stress is evidenced by the fact that overexpression of pyrabactin resistance-like abscisic acid receptors, PYR/PYL/RCARs, enhances drought and cold tolerance in transgenic poplar growing under controlled conditions (Yu *et al.*, 2017). However, these transgenic lines did not show enhanced drought tolerance under natural conditions (Yu *et al.*, 2019), indicating that further research on PYR/PYL/RCARs is needed to determine their potential for biotechnological solutions to drought stress in poplar under changing environments. In Arabidopsis, it is well established that promotion of drought stress involves the expression of genes that contain ABA-responsive elements (ABREs) in their promoters, which are induced by ABA through the action of ABA-responsive element binding (AREB) TFs. During drought stress, changes in the acetylation state of the residue Lys9 of histone H3 modulates the methylation status of several ABREs, allowing the transcription of several ABA-responsive genes (Kim *et al.*, 2012). In *P. trichocarpa*, it has been shown that the AREB1-2 TF binds to ABREs of NO APICAL MERISTEM (NAC) genes and recruits histone acetyltransferase units that enable histone acetylation and the enrichment of RNA-polymerase II at the NAC promoters, thus stimulating transcription of the NAC genes and enhancing drought tolerance (Li *et al.*, 2019). In addition, AREB3 overexpressors of *P. tremula* × *tremuloides* show reduced growth under well-watered conditions and a relative increase in root biomass under drought stress (Yu *et al.*, 2019).

Most of the above information on stress signaling in trees is based on studies of possible functional homology between woody plants and model annuals such as Arabidopsis. There is increasing experimental evidence to indicate that common components such as STZ1 and ABA are able to act in several stress responses. However, their roles in possible interconnections and crosstalk between drought and temperature stresses are unknown. Furthermore, the regulation and relevance of these factors in natural environments and under conditions that promote hardening have not yet been tested. A combination of complementary methods that extend experiments to a wider range of taxa and developmental stages will improve our knowledge of the molecular signaling pathways that operate under abiotic stresses in trees. Combined with recent advances in -omics and NGS disciplines, such studies will provide opportunities to expand our knowledge and to detect unique molecular elements and interactions within abiotic stress pathways in forestry species.

## Genetic architecture of stress responses in tree species

Among the different NGS techniques, massively parallel transcriptome sequencing (i.e. RNA-seq) is one of the most used in trees. RNA-seq aims to determine the transcriptional structure of genes and to quantify and compare the expression levels of transcripts between ontogenetic and/or physiological conditions (Wang *et al.*, 2009). Now already in its ‘teenage years’ (Stark *et al.*, 2019), RNA-seq has most often been used for analysing differential gene expression between experimental groups.

Transcriptomic analyses have the potential to provide information that can be used as a template for exploring the functional regulation of physiological traits. A study of *Populus* by Ruttink *et al.* (2007) provides a good example. The authors demonstrated that short days (SDs) alone during bud development are sufficient to induce genes responsive to dehydration, cold, and ABA. Based on transcriptomic data, they proposed that photoperiod, ethylene, and ABA act as major signals in SD-induced bud formation, growth cessation, and dormancy. These findings provided a template that was then used extensively to further explore bud formation and dormancy in deciduous trees (e.g. Hoffman *et al.*, 2010; Kozarewa *et al.*, 2010; Karlberg *et al.*, 2011).

The first RNA-seq studies that investigated gene expression under abiotic stress in trees were conducted in economically important genera such as *Populus* (Peng *et al.*, 2012) and *Eucalyptus* (Villar *et al.*, 2011). RNA-seq studies that have examined temperature and drought stress in tree species and that have been published in the last 5 years are summarized in Table 1. Here, we consider only the sequencing of mature mRNA, not other types such as miRNA (see ‘Stress and epigenetics’, below). For a review of early transcriptome studies of abiotic stresses in forest trees (mostly microarrays and expressed sequence tag/Sanger sequencing) see Harfouche *et al.* (2014).

**Table 1.** RNA-seq studies published in the last 5 years involving temperature and drought stress in tree species

Species (family)	NCBI BioProject	Sequencing device	Abiotic condition	Tissue	Reference
*Pinus halepensis* (Pinaceae)	PRJNA399618	Illumina HiSeq 2500	Drought	Leaves	Fox *et al.* (2018)
*Pseudotsuga menziesii* (Pinaceae)	PRJNA296922	Illumina HiSeq 2000	Temperature, drought	Leaves	Hess *et al.* (2016)
*Abies alba* (Pinaceae)	PRJNA266095	Illumina HiSeq 2000	Drought	Cotyledons	Behringer *et al.* (2015)
*Platycladus orientalis* (Cupressaceae)	PRJNA318568	Illumina HiSeq 4000	Drought	Roots	Zhang *et al.* (2016)
*Ammopiptanthus mongolicus* (Fabaceae)	PRJNA158883	Illumina HiSeq 2000	Drought	Leaves	Gao *et al.* (2015)
*Prunus dulcis* (Rosaceae)	PRJNA244904	Illumina HiSeq 2000	Freezing	Anther, ovary	Mousavi *et al.* (2014)
*Prunus persica* (Rosaceae)	PRJEB12334	Illumina HiSeq 2000	Drought	Roots, leaves	Ksouri *et al.* (2016)
*Pyrus betulaefolia* (Rosaceae)	–	Illumina/Solexa GAIIx	Drought	Leaves	Li *et al.* (2016)
*Hippophae rhamnoides* (Elaeagnaceae)	PRJNA449450	Illumina HiSeq 4000	Drought	Leaves	Ye *et al.* (2018)
*Broussonetia papyrifera* (Moraceae)	PRJNA219364	Illumina/Solexa GAIIx	Cold	Leaves	Peng *et al.* (2015)
*Quercus lobata* (Fagaceae)	PRJNA357098	Illumina HiSeq 2000	Drought	Leaves	Gugger *et al.* (2017)
*Quercus suber* (Fagaceae)	PRJNA275997	Roche-454 GS FLX Titanium	Drought	Roots	Magalhães *et al.* (2016)
*Populus davidiana* (Salicaceae)	PRJNA384191, PRJNA384193, PRJNA384196, PRJNA384197, PRJNA384198, PRJNA384199	Illumina HiSeq 2500	Drought	Leaves	Mun *et al.* (2017)
*Populus simonii* (Salicaceae)	PRJNA299038	Illumina HiSeq 2000 / HiSeq 2500	Temperature, drought	Leaves, roots	Jia *et al.* (2017)
*Populus trichocarpa* (Salicaceae)	PRJNA227790	Illumina HiSeq 2000	Drought	Leaves	Tang *et al.* (2015)
	PRJNA431471		Cold (and others)	Leaves	Xing *et al.* (2018)
	PRJEB19784		Temperature, drought	Leaves, roots, xylem	Filichkin *et al.* (2018)
*Bombax ceiba* (Malvaceae)	–	Illumina HiSeq 2500	Drought	Leaves	Zhou *et al.* (2015)
*Santalum album* (Santalaceae)	PRJNA320980	Illumina HiSeq 2000	Cold	Leaves	Zhang *et al.* (2017b)
*Camellia sinensis* (Theaceae)	PRJNA297732	Illumina HiSeq 2000	Drought	Leaves	Liu *et al.* (2016)
	PRJEB11522	Illumina HiSeq 2500			Zhang *et al.* (2017a)
*Fraxinus pennsylvanica* (Oleaceae)	PRJNA273266	Illumina MiSeq/HiSeq 2000	Temperature, drought (and others)	Leaves, roots	Lane *et al.* (2016)
*Olea europaea* (Oleaceae)	PRJNA256033	Illumina HiSeq 1000	Cold	Leaves	Leyva-Pérez *et al.* (2015)
	PRJNA272494	Illumina/Solexa GAIIx			Guerra *et al.* (2015)

Recent RNA-seq studies have been carried out in both gymnosperms (four species, two families) and angiosperms (17 species, 11 families) using juvenile plants (Table 1). The economically important species include both timber trees (such as *Pseudotsuga menziesii*, Douglas fir) and non-timber trees (such as *Camellia sinensis*, tea). There are also species that are important as unique constituents of isolated or extreme environments, such as *Ammopiptanthus mongolicus*, an endemic legume species of the eastern Asian deserts, and *Quercus lobata* (valley oak), which grows exclusively in California valleys and foothills. For most of these species, no genomic resources were available prior to the publication of the studies reviewed here. Consequently, the majority of the studies listed in Table 1 are of an exploratory nature and they generally do not deal with particular genes or gene families, or with the regulation of gene expression or transcript variants, and instead they examine general processes involved in the whole-organism response to the environment. As an exception, the availability of the *P. trichocarpa* genome has allowed some authors to focus on the analysis of particular genes (Mun *et al.*, 2017; Xing *et al.*, 2018) or on alternative splicing (Filichkin *et al.*, 2018).

The majority of studies have applied drought as the stress factor and measured its effect on mature leaf tissues; the other commonly examined tissue was the roots. With regards to data availability, most studies provide a link to the NCBI BioProject that contains the raw reads and other relevant data for the reported experiments. This not only fosters transparency and replicability, it also provides an opportunity for meta-analysis and potential new scientific discoveries (Mangul *et al.*, 2019).

Even though there is a huge variety of bioinformatics softwares available online (Dozmorov, 2018; Russell *et al.*, 2018), some programs are used more often than others and have been widely adopted by the research community (Wren, 2016), and these are summarized in Supplementary Table S1 at *JXB* online. Summaries of Gene Ontology terms (GO; Ashburner *et al.*, 2000) and Kyoto Encyclopedia of Genes and Genomes pathways (KEGG; Kanehisa and Goto, 2000) are provided in Table 2, and these link specific genes with higher-order characteristics such as metabolic pathways or biological processes of genes that are over- or under-represented in the RNA-seq studies that we review in this section. As we have already noted, some stresses and tissues have received more attention than others, and this creates some gaps that hinder a thorough comparison between studies. For example, there are no KEGG pathway enrichment analyses for heat stress or for root tissue studied under high or low temperature stress.

**Table 2.** Representative differentially expressed GO terms and KEGG pathways in RNA-seq studies of tree species

Response	Regulation	GO Term/KEGG Pathway	Species (stress)
Photosynthesis	Down	Photosynthesis (GO:0015979)	*Olea europaea* (cold), *Pseudotsuga menziesii* (heat), *Quercus lobata* (drought)
		Photosynthesis (ko00195)	*Camellia sinensis* (drought), *Pyrus betulaefolia* (drought)
		Carbon fixation in photosynthetic organisms (ko00710)	*Santalum album* (cold), *Pyrus betulaefolia* (drought)
ROS scavenging	Up	Secondary metabolic process (GO:0019748)	*Abies alba* (drought), *Ammopiptanthus mongolicus* (drought)
		Steroid biosynthetic process (GO:0006694)	*Populus trichocarpa* (drought)
		Peroxisome organization (GO:0007031)	*Pseudotsuga menziesii* (drought)
		Tropane, piperidine, and pyridine alkaloid biosynthesis (ko00960)	*Pyrus betulaefolia* (drought)
		Diterpenoid biosynthesis (ko00904)	*Populus trichocarpa* (drought), *Santalum album* (cold)
		Flavonoid biosynthesis (ko00941)	*Camellia sinensis* (drought), *Populus trichocarpa* (drought)
		Phenylpropanoid biosynthesis (ko00940)	*Platycladus orientalis* (drought)
		Isoprenoid biosynthetic process (GO:0008299)	*Prunus dulcis* (freezing)
Stress (general)	Up	Response to abiotic stimulus (GO:0009628)	*Fraxinus pennsylvanica* (cold, drought), *Santalum album* (cold)
		Response to stress (GO:0006950)	*Ammopiptanthus mongolicus* (drought), *Fraxinus pennsylvanica* (heat), *Quercus lobata* (drought), *Populus trichocarpa* (drought), *Prunus persica* (drought), *Platycladus orientalis* (drought)*, Bombax ceiba* (drought)
		Cellular response to stress (GO:0033554)	*Pseudotsuga menziesii* (heat)
Stress (specific)	Up	Cold acclimation (GO:0009631)	*Olea europaea* (cold)
		Response to cold (GO:0009409)	*Cupressus sempervirens* (cold)
		Response to heat (GO:0009408)	*Pseudotsuga menziesii* (heat)
		Heat acclimation (GO:0010286)	*Pseudotsuga menziesii* (heat)
		Response to osmotic stress (GO:0006970)	*Abies alba* (drought)
		Response to water (GO:0009415)	*Hippophae rhamnoides* (drought)
		Response to water deprivation (GO:0009414)	*Abies alba* (drought), *Bombax ceiba* (drought)

A common feature across all stresses (drought, cold, or heat) in leaf tissue is the down-regulation of photosynthesis. Flower sexual tissues, which are not usually involved in photosynthesis, predictably do not feature down-regulation of photosynthetic processes or pathways in *Prunus dulcis*. The repression of photosynthesis indicates a drastic rearrangement of resource allocation in plants under stress.

A well-known effect of abiotic stress in plants is the production of ROS, which can eventually oxidize lipids, proteins, and DNA, and thereby trigger cell death (Akula and Ravishankar, 2011; see previous section). To prevent this, plants accumulate antioxidant compounds such as flavonoids, alkaloids, or terpenoids (isoprenoids), as well as brassinosteroids in response to various stresses and stress combinations (Akula and Ravishankar, 2011; Bartwal *et al.*, 2013; Nakabayashi and Saito, 2015; Choudhury *et al.*, 2017). This is reflected in the RNA-seq studies reviewed here, across different stress types and also tissues (flowers of *Prunus dulcis*, and roots of *P. persica* and *Platycladus orientalis*; Table 2).

On a more specific level, there are several genes or group of genes that are commonly reported as being up- or down-regulated in the studies reviewed here. These include CBF TFs, which belong to the AP2/ERF family and are activated under cold stress (see also previous section). Homologs of CBF genes are up-regulated in response to cold stress in *O. europaea* and *S. album* (Table 2). Several other TFs of the AP2/ERF family are also differentially expressed in response to drought and/or temperature stress in *Pseudotsuga menziesii*, *Prunus dulcis*, *Platycladus orientalis*, *Ammopiptanthus mongolicus*, *Camelia sinensis*, *Hippophae rhamnoides*, *Quercus suber*, and *Populus davidiana*. Other important families of TFs that are notably present in over- or under-represented genes across different species are WRKYs, HSFs, zinc-finger CCCH-types, and NACs (see previous section).

Proteins that act as chaperones, such as Hsps or dehydrins (members of the late embryogenesis abundant, LEA, family) are up-regulated in response to all the types of stresses reviewed here in *Abies alba*, *Cupressus sempervirens*, *Pinus halepensis*, *Prunus persica*, *Quercus suber*, *Q. lobata*, and *Pseudoptsuga menziesii*. The proteins of both the Hsp and LEA families contribute to stabilizing proteins in the process of denaturation, which is common to several types of abiotic stress (Close, 1996; Feder and Hofmann, 1999), and thus they increase stress tolerance.

With regards to hormone pathways, the results are consistent across stressors and species for some hormones but not for others. The signaling pathways that are consistently up-regulated are those of jasmonic acid (JA), brassinosteroids (BRs), and ABA. In contrast, salicylic acid (SA) transduction is reported to be down-regulated in *Ammopiptanthus mongolicus*, *Bombax ceiba*, and *Pinus halepensis*. Interestingly, auxin signal transduction appears as up-regulated in response to stress in some species (*A. mongolicus*, *B. ceiba*, *Santalum album*, *Hippophae rhamnoides*) but down-regulated in others (*Camellia sinensis*, *Populus simonii*, *Pinus halepensis*). In *A. mongolicus*, *C. sinensis*, and *B. ceiba*, the opposite pattern is observed with ethylene transduction: it is up-regulated when auxin is down-regulated, and vice versa. These results are indicative of a complex transcriptional landscape in response to abiotic stress, and in particular they show highly variable interactions between different hormone signal transduction pathways.

## Stress and epigenetics

The term epigenetics summarizes different mechanisms that change the nucleosome structure (such as DNA methylation and histone modifications) and thereby influence gene expression (Lämke and Bäurle, 2017). As long-lived organisms, epigenetic mechanisms in trees might provide a fast way for reacting to environmental changes, including abiotic stresses (Bräutigam *et al.*, 2013). When considering the role of epigenetic regulation in trees in responses to drought and thermal stress, most studies have dealt with DNA methylation. The first studies were limited to measuring global DNA methylation, where the proportion of methylated bases across the genome was determined by HPLC assays (Alonso *et al.*, 2015). This was followed by studies that used methylation-sensitive restriction enzymes combined with an amplified fragment length polymorphism approach (methylation-sensitive amplified polymorphism, MSAP; Reyna-López *et al.*, 1997), which yielded anonymous data on changes of methylation at a few hundred sites. These older methods for functional analyses have limited informative value, and the more detailed bisulfite sequencing technique has become the method of choice in recent years (Richards *et al.*, 2017). Depending on the genomic and financial resources available, either the entire genome can be sequenced (whole-genome bisulfite sequencing, WGBS) or the genome size can be reduced in a targeted (e.g. expressed regions of the genome; Lee *et al.*, 2011) or untargeted manner (reduced-representation bisulfite sequencing, RRBS; Paun *et al.*, 2019). Given that the DNA sequence is of reduced complexity after bisulfite treatment, the mapping and identification of differentially methylated positions (DMPs) and regions (DMRs) involves a number of bioinformatic challenges, which have recently been summarized by (Richards *et al.*, (2017). Nevertheless, because of the challenges involved in conducting bisulfite sequencing studies, the number of sequence-based studies that provide in-depth insights into the role of epigenetics in trees is still limited. A total of 50 studies were listed in a recent review, including studies on miRNAs that classically do not fall under the term epigenetics (Sow *et al.*, 2018). Here, we adopt the same rationale that due to the important role of miRNAs in post-transcriptional regulation of many epigenetic genes, miRNAs can be considered as important players in the epigenetic machinery. Of the 50 studies listed in the review, only five were based on WGBS, while the majority were still based on MSAPs.

The earliest WBGS study in poplars showed that methylation patterns change specifically in many TFs following drought stress treatment (Liang *et al.*, 2014). Methylation changes lead to decreases in gene expression if they are close to the transcription start site, while a change of methylation in the gene body is linked to higher gene expression. Liang *et al.* (2014) were also able to confirm an important role of transposable elements (TEs) located in promoter regions and in gene bodies of TFs related to plant stress resistance. A change in methylation of TEs is observed in well-watered versus drought-stressed *P. trichocarpa* plants, which indicates that TEs are involved in regulating gene expression (Liang *et al.*, 2014). Likewise, a study on apple (*Malus domesticus*) showed abundant demethylation of TEs under drought stress (Xu *et al.*, 2018). Relating their findings to studies on maize and *Arabidopsis*, these authors speculated that dynamic demethylation alterations to transposons and proximal genes are indeed related to drought stress. In another study in poplar, hypomethylation was observed under drought stress, mainly in gene bodies in cells of the somatic apical meristem (SAM) (Lafon-Placette *et al.*, 2018), followed by hypermethylation after re-watering. The opposite effect was observed for TEs, indicating a highly dynamic response to water status. While no consistent changes in methylation and expression could be observed for the majority of genes, the authors found that a number of hormone-responsive genes were hypomethylated and showed down-regulation during re-watering, suggesting crosstalk between DNA methylation and polycomb complexes (enzymes that repress gene transcription via histone modifications; Mozgova *et al.*, 2015) under drought stress, and emphasizing the role of methylation in regulating drought-induced hormone pathways including genes activated by cytokinin, jasmonate, salicylic acid, and ABA-, or genes repressed by auxin and ethylene. For example, in Arabidopsis, overexpression of constitutive active DREB2A (a member of a gene family linked to plant resistance to heat and drought stress) results in significantly increased drought tolerance (Sakuma *et al.*, 2006). In apple trees, there are differences in expression of DREB2A and in methylation in response to drought stress between *M.* × *domestica* and its wild relative *M. prunifolia*, which is known to be more drought tolerant than the cultivated species (Li *et al.*, 2019). This study showed that the methylation level of the promoter region of DREB2A in *M. prunifolia* was significantly reduced, and the expression of the gene was increased 100-fold under drought stress, while an increase of expression only 16-fold could be observed in *M.* × *domestica*. The authors concluded that regulation of methylation in this promoter region contributes to the drought resistance of *M. prunifolia*.

In contrast to tree species such as poplar and apple that have relatively well-annotated genomes, studies in conifers are still challenging due to their large genomes for which only highly fragmented studies are available (Nystedt *et al.*, 2013; Mosca *et al.*, 2019). However, epigenetic phenomena have been studied extensively in Norway spruce (Yakovlev *et al.*, 2012; Heer *et al.*, 2018). For example, Yakovlev *et al.* (2012) investigated the expression of genes involved in epigenetic regulation and ‘memory formation’ under temperature regimes responsible for inducing previously observed epitypes. They found that genes involved in DNA and histone methylation such as *METHYLTRANSFERASE1* (*MET1*) and *HISTONE DEMETHYLTRANSFERASE* (*HDMT*) were prominent among the differentially expressed genes (DEGs), indicating that epigenetics mechanisms are involved in the response to heat stress in conifers as well.

As noted above, miRNAs play a crucial role in the regulation of epigenetic mechanisms, and seem to be important for the response of plants to drought and heat stress. miRNAs regulate gene expression through the post-transcriptional silencing of complementary mRNA (Liu *et al.*, 2015). Changes in miRNA expression have been reported in tree species, for example in response to heat stress in *Betula luminifera* (Pan *et al.*, 2017) and in several poplar species (Li *et al.*, 2011; Chen *et al.*, 2012), and also in response to drought stress (Ren *et al.*, 2012; Shuai *et al.*, 2013).

Overall, there are a number of studies that indicate a role of epigenetic mechanisms for acclimation to drought and heat in trees. However, the limited number of genomically and experimentally sophisticated studies leaves much to be explored.

## Genetic footprints of tree adaptation to stressful environments

Adaptation at a local scale is a crucial evolutionary process that allows plants to grow better in their native compared to non-native habitat (Martins *et al.*, 2018) and it shapes the genetic diversity of the species in response to geographically varying selection pressures (Tiffin and Ross-Ibarra, 2014). The geographic patterns displayed by species offer a unique study system to identify genome regions involved in the adaptation process across their distribution range. The action of natural selection along gradually changing environments leads to genetic differentiation between adjacent populations (Linhart and Grant, 1996). However, when a collinear relationship of geography with climate variables exists, the genetic differences caused by ecologically divergent habitats (‘isolation by environment’) may result in similar genetic patterns to those caused by isolation by geography (‘isolation by distance’) (Nadeau *et al.*, 2016). Climatic oscillations of the Pleistocene promoted demographic processes that are still evident in the genetic footprints of tree populations (Magri *et al.*, 2006; Turchetto-Zolet *et al.*, 2013); thus, ancient marks of past times are imprinted in their genomes. Genetic clines generated as a result of isolation, limited gene flow, or the presence of variants restricted to particular areas support the fact that past signatures might have a key role in the current adaptive response of some populations (Grivet *et al.*, 2011; Modesto *et al.*, 2014; Mosca *et al.*, 2016). Such considerations must be *a priori* taken into account in order to obtain more reliable identification of adaptive alleles, in order to avoid confusing the effects of demographic population changes with those of local adaptation (Bragg *et al.*, 2015).

In recent decades, the challenge for forest scientists has been to develop technological and analytical approaches that allow the elucidation the genomic regions that contribute to the adaptation of trees to certain climatic conditions. The release of the first complete genome in a tree species (*P. tricocharpa*; Tuskan *et al.*, 2006) was highly relevant since it became a reference for the functional annotations of new genomic resources that would begin to be generated in many other trees. The advent of genome sequencing (see above) represents a great opportunity for expanding the methods used in adaptation studies in tree species along their natural distribution.

Most of the early research that looked for evidence of adaptive loci at the DNA level used candidate gene approaches that were based on molecular markers associated with them. At the same time, approximations were developed that were known as forward genetics, first quantitative trait loci (QTLs) and then genome-wide association studies (GWAS; Pardo-Diaz *et al.*, 2015, Fetter *et al.*, 2017). The advent of genome scanning techniques meant that the identification of selection signatures could be done with or without previous knowledge of the target genes (Lotterhos and Whitlock, 2015), which marked a clear difference with candidate gene studies. Here, we focus on studies that have reported adaptive variation for candidate genes and genome-scan markers in economically relevant woody genera within their natural range.

Whether they have concerned individual loci or hundreds of markers, most studies that have aimed to identify the genetic determinants of local adaptation of trees growing in a climate gradient have implemented either one or both of the following methods: statistical tools that seek to define the adaptive differentiation based on departures (outliers) of the fixation index (*F*_ST_) from a theoretically neutral distribution (Lewontin and Krakauer, 1973); and/or statistical tools that test genotype–environment correlations (reviewed by Rellstab *et al.*, 2015). The first evidence of adaptive loci involved in drought stress responses and tolerance to extreme temperatures were based on outlier detection using BayeScan (Foll and Gaggiotti, 2008) and LOSITAN (Antao *et al.*, 2008). Many functional gene categories (e.g. oxidoreductases, TFs, chaperone genes) have been highlighted as key responses in *Fagus sylvatica* (Carsjens *et al.*, 2014; Modesto *et al.*, 2014; Krajmerová *et al.*, 2017), several *Quercus* species (Lind-Riehl and Gailing, 2017; Pina-Martins *et al.*, 2019), and *Abies alba* (Roschanski *et al.*, 2016), among others.

The nature of selection can be detected by statistical approaches based on genotype–environment association (GEA) data (Endler, 1986; Lotterhos and Whitlock, 2015). In this case, detailed environmental information at the scale of the site is a key requirement to define the association of allelic variants with climate variables. Numerous statistical programs have been reported in the literature, but only a few consider the population structure in the test and these involve Bayesian simulation (Bayenv2; Coop *et al.*, 2010; Günther and Coop, 2013) or Latent Factor Mixed Models (LFMMs; Frichot *et al.*, 2013); these are available as packages of the R statistical software (lfmm, LEA). Using GEA data, it has been confirmed for several tree species that gene variation at dehydrins and histones is associated with specific environmental variables (Dillon *et al.*, 2014; Krajmerová *et al.*, 2017; Müller and Gailing, 2019). Stress-inducible dehydrins belonging to the late-embryogenesis abundant (LEA) protein family are regulated by TFs binding to specific responsive elements such us ABRE, CRT/DRE/LTRE, MYB, and MYC (Hanin *et al.*, 2011). Dehydrins are a major group of versatile proteins that play a role in many oxidative stress responses, for example they participate in the protection of membrane integrity (Yu *et al.*, 2018; see above, and Fig. 1). In addition, histone modifications, either at the nucleotide level or as epigenetic changes, act like an internal ‘memory’ for information storage in the plant stress response (Jaskiewicz *et al.*, 2011).

A new generation of studies that aim to detect signals of local adaptation are ‘genome-wide’ approximations. These involve major sampling of loci that are regarded as ‘hot spots’ regions responsible for the global adaptive responses of populations across their environments (as summarized by Hoban *et al.*, 2016). Genome scanning associated with environmental clines is then possible and the inferences gain in accuracy as the crucial selecting pressures (e.g. extreme temperature, drought, aridity) that shape species-specific variation are uncovered. Using this approach, several studies have been able to identify sets of loci associated with specific climate variables, such as maximum temperature and annual precipitation in *Pinus cembra* and *P. mugo* (Mosca *et al.*, 2016), winter drought, relative humidity, and vapor water deficit in *Abies alba* (Roschanski *et al.*, 2016), the aridity index in *Eucalyptus* (Steane *et al.*, 2017), mean annual temperature and precipitation in *P. trichocarpa* (Evans *et al.*, 2014), and at least 12 climate variables in *P. trichocarpa* including mean coldest-month temperature, extreme minimum temperature over a 30-year period, and mean annual precipitation (Geraldes *et al.*, 2014).

In agreement with the stress-response pathways that we considered in the section on ‘Genetic architecture’ (above), the gene functional categories of woody species most frequently associated with environmental variables include TFs such as CBFs (Geraldes *et al.*, 2014; Meireles *et al.*, 2017), zinc-finger domain-containing proteins (Evans *et al.*, 2014; Geraldes *et al.*, 2014), antioxidant molecules acting in the detoxification of ROS (Ruiz Daniels et al., 2017), protein phosphatase 2C (Carsjens *et al.*, 2014; Evans *et al.*, 2014; Cuervo-Alarcon *et al.*, 2018), and class 1 small Hsps (Austerlitz *et al.*, 2004; Modesto *et al.*, 2014; Mosca *et al.*, 2016; Sork *et al.*, 2016) (Supplementary Table S2). Interestingly, these studies report the relevance of several molecules produced either at the beginning or at the end of a long response pathway. In the case of the former, they include regulators of various ABA-dependent or independent transduction signals, whilst in the latter, final products such as detoxification or stabilizing proteins are relevant since they accomplish key roles in overall cellular protection systems that allow cellular homeostasis against several stresses to be maintained (Vaseva *et al.*, 2012).

In summary, the genetic footprint of tree adaptation produced in response to abiotic stress (extreme temperatures and drought) is complex, with species-dependent interactions among numerous ‘hot-spots’ distributed across the genome. An overview needs to be taken that includes the various approaches considered here together with other resources such as expression profiles and epigenomes in order to thoroughly understand the response of woody plants to climate change.

## Conclusions and future prospects

The ability of forests to respond to global change is of utmost importance for the future of the planet. Trees are facing rapid changes in temperature and precipitation (Joyce and Rehfeldt, 2013) that are affecting forest productivity, survival, and the distribution of species (Birdsey and Pan, 2011; Morin *et al.*, 2018). In this context, gaining knowledge related to the molecular bases of responses to abiotic stress is an urgent requirement that will contribute to the detection, and potentially to the generation, of genotypes that are more resistant to abiotic stress. It is worth noting the relative scarcity of molecular information that is available in the literature in relation to responses to heat stress in trees, one of the most important abiotic stresses in the context of global warming.

Our present knowledge indicates that responses to drought and temperature stresses encompass a wide spectrum of molecular responses, including the actions of MAPK cascades, calcium signaling components, ubiquitin ligases, and several types of TFs (Fig. 1). These different pathways trigger processes such as the epigenetic regulation of hormone-responsive genes, the activity of TEs and TFs, and the reprogramming of a large proportion of the transcriptome. Consistent with physiological studies, transcriptome rearrangements include changes in the expression of genes related to metabolic processes such as photosynthesis, ROS homeostasis, secondary metabolism, and hormone signaling and responses. It is important to note that several genes, for example those coding for CBFs, ABA response, Hsps, and zinc finger-type TFs, have been found to be linked to abiotic stress by functional, transcriptomic, and population genomics studies across different species, indicating their strong relevance in responses and adaptations to abiotic stress in trees. For example, CBFs are up-regulated by MYB TFs during chilling in apple (Fig. 1), and transcriptomic studies have shown their expression is also up-regulated by cold in *Olea europea* and *Santalum album*. In *Quercus*, the inducer of CBF expression (ICE) is under selection pressure in natural populations (Supplementary Table S2). These studies provide strong evidence that the C-repeat/DREB binding factors regulon is a crucial module for cold responses in trees.

Among the -omics, transcriptomic studies are the most abundant in tree species. This is to be expected, given that advances in NGS technologies, their relatively low costs, and the increasing availability of open access bioinformatics tools for the analysis of transcriptome-derived sequences all allow the generation of huge amounts of genetic information, even in the absence of reference genomes. As a result, transcriptomics usually constitutes the first approach towards molecular studies in woody plants. However, it is important to note that because of the variabilities in the methods used to induce stress, in the tissues sampled, and in the bioinformatics tools used to generate and analyse the data (Table 1, Supplementary Table S1), the interpretation of differences between studies must be performed with caution, as they could constitute methodological artefacts. In contrast with transcriptomic studies, molecular information derived from epigenetics is scarce. Given the important role that epigenetic processes may have in the adaptation of long-living organisms to changes in the environment (Bräutigam *et al.*, 2013), epigenetics studies related to the responses to abiotic stress in trees must be a priority for future research.

Despite the large amount of data available, it is important to note that abiotic stress studies in trees have been relatively fragmented in comparison to Arabidopsis and crop species. For example, it is difficult to draw a sequential signaling pathway from receptors to effectors, and the identities of specific sensors that trigger abiotic stress responses remain elusive. We still do not know whether many of the mechanisms that have been described are conserved between angiosperms and gymnosperms, and information regarding tissue-specific signaling and resistances to stress is scarce. For instance, in stems, living cells of the parenchyma and pith are often less resistant to low temperatures than those of the phloem, cortex, and epidermis (reviewed by Weiser, 1970), and in conifer species tracheids are more abundant and have thinner walls when they are produced during well-watered periods compared to those produced during drought (Eldhuset *et al.*, 2013; Xu *et al.*, 2014). The combined use of physiological and metabolomics approaches together with new molecular technologies such as single-cell RNA sequencing may help future studies examining the pathways that operate at a tissue-specific level in trees, which are highly relevant for their stress responses. This information is of utmost importance for the development of biotechnological tools for genetic engineering of trees with increased stress tolerance. Our ability to translate our current knowledge of drought and temperature responses into biotechnological solutions for increasing forest productivity and conservation under future predicted climatic scenarios remains a challenging prospect. For example, one promising study has used transgenic lines to show that the overexpression of genes related to ABA signaling and responses results in increased resistance to drought in poplar (Yu *et al.*, 2017). However, we still do not know the way in which changes in the expression of such molecular components may affect growth and development in natural conditions. For example, will we be able to decouple molecular processes that influence stress tolerance from those related to reduction in growth? In other words, will we be able to take advantage of the positive outcomes of an enhanced ABA response in terms of stress resistance whilst minimizing its consequences on phenology and growth? This is a very important consideration in tree species that are used as crops for commercial purposes such as *Eucalyptus*, which is the most-planted hardwood genus across the world due to is exceptionally rapid growth and high wood quality, but which is limited in its productivity and distribution by low temperatures (Myburg *et al.*, 2007). Another challenging point is that generally the responses of plants to a combination of stresses cannot easily be predicted from the responses to individual stresses alone. In the natural environment, trees usually experience different stresses simultaneously, but most of the molecular information on abiotic stress is based on their responses to a single stress factor, for example to drought or a specific temperature stress. Recent transcriptomic studies have indicated that the response of poplar to a combination of drought and high temperatures, which are often present simultaneously in the natural environment, is quite different to the responses to only drought or heat (Jia *et al.*, 2017). Once the basic information related to the molecular pathways involved in the response of trees to individual stresses becomes clearer, research should move on to the integration of multiple environmental stresses across the tree life-cycle. In addition, the combination of laboratory work with outdoor experiments across environmental gradients would constitute a powerful approach to test the responses of particular genotypes to multiple stresses and to extreme conditions in the natural environment.

Finally, it is important to note that linking our understanding of biological processes at different scales is a major conceptual challenge in biology, and one that becomes even more complex as the result of the differences that exist between the methods applied in different research projects (Zardilis *et al.*, 2019). In Arabidopsis, the application of systems biology approaches has allowed the identification and association of gene expression patterns to physiological characteristics, thereby providing mechanistic insights into genome functioning under abiotic stress conditions (Weston *et al.*, 2008). Furthermore, the combination of plant modelling with growth and phenology studies in Arabidopsis has allowed the creation of whole-life-cycle multi-models that can simulate various genotype × environment scenarios at the population level (Zardilis *et al.*, 2019). Recently, the ‘crops *in silico*’ initiative has been proposed, with the claim that multi-scale models have the potential to fill in missing mechanistic details and to generate new hypotheses that will prioritize the direction of bio-engineering efforts in plant science (Millar *et al.*, 2019). Will we be able to orientate studies in trees in order to create developmental models that match the biological characteristics of these long-living organisms, and be able to describe their responses to changes in the environment from a holistic point of view? Recent work by Chateigner *et al.* (2019, Preprint) that proposes the use of omnic models, building co-expression networks to define core and peripheral genes and predict their contribution to phenotypes, constitutes a promising start to this sort of work in trees. The development of such holistic approaches will significantly improve our understanding of the molecular networks that underlie the abiotic stress responses at the whole-plant and organ level, and will increase our ability to design strategies to predict and potentially promote tree fitness under changing climates.

## Supplementary data

Supplementary data are available at *JXB* online.

Table S1. Bioinformatic tools used in RNA-seq studies of tree species.

Table S2. Description and functional annotation of candidate genes related to drought and temperature stress.

erz532_suppl_Supplementary_Table_S1-S2

## References

[CIT0001] Aharoni A, Dixit S, Jetter R, Thoenes E, van Arkel G, Pereira A 2004 The SHINE clade of AP2 domain transcription factors activates wax biosynthesis, alters cuticle properties, and confers drought tolerance when overexpressed in *Arabidopsis*. The Plant Cell 16, 2463–2480. 15319479 10.1105/tpc.104.022897 PMC520946

[CIT0002] Akula R, Ravishankar GA 2011 Influence of abiotic stress signals on secondary metabolites in plants. Plant Signaling & Behavior 6, 1720–1731. 22041989 10.4161/psb.6.11.17613 PMC3329344

[CIT0003] Allen DJ, Ratner K, Giller YE, Gussakovsky EE, Shahak Y, Ort DR 2000 An overnight chill induces a delayed inhibition of photosynthesis at midday in mango (*Mangifera indica* L.). Journal of Experimental Botany 51, 1893–1902. 11113167 10.1093/jexbot/51.352.1893

[CIT0004] Alonso C, Pérez R, Bazaga P, Herrera CM 2015 Global DNA cytosine methylation as an evolving trait: phylogenetic signal and correlated evolution with genome size in angiosperms. Frontiers in Genetics 6, 4. 25688257 10.3389/fgene.2015.00004 PMC4310347

[CIT0005] Antao T, Lopes A, Lopes RJ, Beja-Pereira A, Luikart G 2008 LOSITAN: a workbench to detect molecular adaptation based on a *F*_*st*_-outlier method. BMC Bioinformatics 9, 323. 18662398 10.1186/1471-2105-9-323 PMC2515854

[CIT0006] Ashburner M, Ball CA, Blake JA, et al 2000 Gene Ontology: tool for the unification of biology. Nature Genetics 25, 25–29. 10802651 10.1038/75556 PMC3037419

[CIT0007] Austerlitz F, Dick CW, Dutech C, Klein EK, Oddou-Muratorio S, Smouse PE, Sork VL 2004 Using genetic markers to estimate the pollen dispersal curve. Molecular Ecology 13, 937–954. 15012767 10.1111/j.1365-294x.2004.02100.x

[CIT0008] Bartwal A, Mall R, Lohani P, Guru SK, Arora S 2013 Role of secondary metabolites and brassinosteroids in plant defense against environmental stresses. Journal of Plant Growth Regulation 32, 216–232.

[CIT0009] Behringer D, Zimmermann H, Ziegenhagen B, Liepelt S 2015 Differential gene expression reveals candidate genes for drought stress response in *Abies alba* (Pinaceae). PloS ONE 10, e0124564. 25924061 10.1371/journal.pone.0124564 PMC4414588

[CIT0010] Benedict C, Skinner JS, Meng R, Chang Y, Bhalerao R, Huner NP, Finn CE, Chen TH, Hurry V 2006 The CBF1-dependent low temperature signalling pathway, regulon and increase in freeze tolerance are conserved in *Populus* spp. Plant, Cell & Environment 29, 1259–1272. 10.1111/j.1365-3040.2006.01505.x 17080948

[CIT0011] Birdsey R, Pan Y 2011 Drought and dead trees. Nature Climate Change 1, 444–445.

[CIT0012] Bjornson M, Dandekar A, Dehesh K 2016 Determinants of timing and amplitude in the plant general stress response. Journal of Integrative Plant Biology 58, 119–126. 26108530 10.1111/jipb.12373

[CIT0013] Bragg JG, Supple MA, Andrew RL, Borevitz JO 2015 Genomic variation across landscapes: insights and applications. New Phytologist 207, 953–967. 25904408 10.1111/nph.13410

[CIT0014] Bräutigam K, Vining KJ, Lafon-Placette C, et al 2013 Epigenetic regulation of adaptive responses of forest tree species to the environment. Ecology and Evolution 3, 399–415. 23467802 10.1002/ece3.461 PMC3586649

[CIT0015] Carsjens C, Nguyen Ngoc Q, Guzy J, Knutzen F, Meier IC, Müller M, Finkeldey R, Leuschner C, Polle A 2014 Intra-specific variations in expression of stress-related genes in beech progenies are stronger than drought-induced responses. Tree Physiology 34, 1348–1361. 25430883 10.1093/treephys/tpu093

[CIT0016] Chateigner A, Lesage-Descauses M-C, Rogier O, et al 2019 Gene expression predictions and networks in natural populations supports the omnigenic theory. bioRxiv 523365. [Preprint]. 10.1186/s12864-020-06809-2 PMC7310122 32571208

[CIT0017] Chen J, Xue B, Xia X, Yin W 2013 A novel calcium-dependent protein kinase gene from *Populus euphratica*, confers both drought and cold stress tolerance. Biochemical and Biophysical Research Communications 441, 630–636. 24177011 10.1016/j.bbrc.2013.10.103

[CIT0018] Chen J, Yin W, Xia X 2014 Transcriptome profiles of *Populus euphratica* upon heat shock stress. Current Genomics 15, 326–340. 25435796 10.2174/138920291505141106101835 PMC4245693

[CIT0019] Chen L, Ren Y, Zhang Y, Xu J, Sun F, Zhang Z, Wang Y 2012 Genome-wide identification and expression analysis of heat-responsive and novel microRNAs in *Populus tomentosa*. Gene 504, 160–165. 22634103 10.1016/j.gene.2012.05.034

[CIT0020] Choat B, Brodribb TJ, Brodersen CR, Duursma RA, López R, Medlyn BE 2018 Triggers of tree mortality under drought. Nature 558, 531–539. 29950621 10.1038/s41586-018-0240-x

[CIT0021] Choudhury FK, Rivero RM, Blumwald E, Mittler R 2017 Reactive oxygen species, abiotic stress and stress combination. The Plant Journal 90, 856–867. 27801967 10.1111/tpj.13299

[CIT0022] Ciais P, Reichstein M, Viovy N, et al 2005 Europe-wide reduction in primary productivity caused by the heat and drought in 2003. Nature 437, 529–533. 16177786 10.1038/nature03972

[CIT0023] Claeys H, Inzé D 2013 The agony of choice: how plants balance growth and survival under water-limiting conditions. Plant Physiology 162, 1768–1779. 23766368 10.1104/pp.113.220921 PMC3729759

[CIT0024] Close TJ 1996 Dehydrins: emergence of a biochemical role of a family of plant dehydration proteins. Physiologia Plantarum 97, 795–803.

[CIT0025] Cooke JE, Eriksson ME, Junttila O 2012 The dynamic nature of bud dormancy in trees: environmental control and molecular mechanisms. Plant, Cell & Environment 35, 1707–1728. 10.1111/j.1365-3040.2012.02552.x 22670814

[CIT0026] Coop G, Witonsky D, Di Rienzo A, Pritchard JK 2010 Using environmental correlations to identify loci underlying local adaptation. Genetics 185, 1411–1423. 20516501 10.1534/genetics.110.114819 PMC2927766

[CIT0027] Crisp PA, Ganguly D, Eichten SR, Borevitz JO, Pogson BJ 2016 Reconsidering plant memory: intersections between stress recovery, RNA turnover, and epigenetics. Science Advances 2, e1501340. 26989783 10.1126/sciadv.1501340 PMC4788475

[CIT0028] Cuervo-Alarcon L, Arend M, Müller M, Sperisen C, Finkeldey R, Krutovsky KV 2018 Genetic variation and signatures of natural selection in populations of European beech (*Fagus sylvatica* L.) along precipitation gradients. Tree Genetics & Genomes 14, 84.

[CIT0029] Dillon S, McEvoy R, Baldwin DS, Rees GN, Parsons Y, Southerton S 2014 Characterisation of adaptive genetic diversity in environmentally contrasted populations of *Eucalyptus camaldulensis* Dehnh. (river red gum). PLoS ONE 9, e103515. 25093589 10.1371/journal.pone.0103515 PMC4122390

[CIT0030] Ding S, Zhang B, Qin F 2015 Arabidopsis RZFP34/CHYR1, a ubiquitin E3 ligase, regulates stomatal movement and drought tolerance via SnRK2.6-mediated phosphorylation. The Plant Cell 27, 3228–3244. 26508764 10.1105/tpc.15.00321 PMC4682294

[CIT0031] Dozmorov MG 2018 GitHub statistics as a measure of the impact of open-source bioinformatics software. Frontiers in Bioengineering and Biotechnology 6, 198. 30619845 10.3389/fbioe.2018.00198 PMC6306043

[CIT0032] Du FK, Petit RJ, Liu JQ 2009 More introgression with less gene flow: chloroplast vs. mitochondrial DNA in the *Picea asperata* complex in China, and comparison with other conifers. Molecular Ecology 18, 1396–1407. 19284474 10.1111/j.1365-294X.2009.04107.x

[CIT0033] Eldhuset TD, Nagy NE, Volařík D, Børja I, Gebauer R, Yakovlev IA, Krokene P 2013 Drought affects tracheid structure, dehydrin expression, and above- and belowground growth in 5-year-old Norway spruce. Plant and Soil 366, 305–320.

[CIT0034] Ellison D, Morris CE, Locatelli B, et al 2017 Trees, forests and water: cool insights for a hot world. Global Environmental Change 43, 51–61.

[CIT0035] Endler J 1986 Natural selection in the wild. Monographs in Population Biology, vol. 21. Princeton, NJ: Princeton University Press.

[CIT0036] Escandón M, Cañal MJ, Pascual J, Pinto G, Correia B, Amaral J, Meijón M 2016 Integrated physiological and hormonal profile of heat-induced thermotolerance in *Pinus radiata*. Tree Physiology 36, 63–77. 26764270 10.1093/treephys/tpv127

[CIT0037] Evans LM, Slavov GT, Rodgers-Melnick E, et al 2014 Population genomics of *Populus trichocarpa* identifies signatures of selection and adaptive trait associations. Nature Genetics 46, 1089–1096. 25151358 10.1038/ng.3075

[CIT0038] Falcone DL, Ogas JP, Somerville CR 2004 Regulation of membrane fatty acid composition by temperature in mutants of *Arabidopsis* with alterations in membrane lipid composition. BMC Plant Biology 4, 17. 15377388 10.1186/1471-2229-4-17 PMC524174

[CIT0039] Feder ME, Hofmann GE 1999 Heat-shock proteins, molecular chaperones, and the stress response: evolutionary and ecological physiology. Annual Review of Physiology 61, 243–282. 10.1146/annurev.physiol.61.1.243 10099689

[CIT0040] Fetter KC, Gugger PF, Keller SR 2017 Landscape genomics of angiosperm trees: from historic roots to discovering new branches of adaptive evolution. In: Groover A, Cronk Q, eds. Comparative and evolutionary genomics of angiosperm trees. Cham, Switzerland: Springer International Publishing, 303–333.

[CIT0041] Filichkin SA, Hamilton M, Dharmawardhana PD, Singh SK, Sullivan C, Ben-Hur A, Reddy ASN, Jaiswal P 2018 Abiotic stresses modulate landscape of poplar transcriptome via alternative splicing, differential intron retention, and isoform ratio switching. Frontiers in Plant Science 9, 5. 29483921 10.3389/fpls.2018.00005 PMC5816337

[CIT0042] Foll M, Gaggiotti O 2008 A genome-scan method to identify selected loci appropriate for both dominant and codominant markers: a Bayesian perspective. Genetics 180, 977–993. 18780740 10.1534/genetics.108.092221 PMC2567396

[CIT0043] Fox H, Doron-Faigenboim A, Kelly G, Bourstein R, Attia Z, Zhou J, Moshe Y, Moshelion M, David-Schwartz R 2018 Transcriptome analysis of *Pinus halepensis* under drought stress and during recovery. Tree Physiology 38, 423–441. 29177514 10.1093/treephys/tpx137 PMC5982726

[CIT0044] Frichot E, Schoville SD, Bouchard G, François O 2013 Testing for associations between loci and environmental gradients using latent factor mixed models. Molecular Biology and Evolution 30, 1687–1699. 23543094 10.1093/molbev/mst063 PMC3684853

[CIT0045] Gao F, Wang J, Wei S, Li Z, Wang N, Li H, Feng J, Li H, Zhou Y, Zhang F 2015 Transcriptomic analysis of drought stress responses in ammopiptanthus mongolicus leaves using the RNA-seq technique. PLoS ONE 10, e0124382. 25923822 10.1371/journal.pone.0124382 PMC4414462

[CIT0046] Geraldes A, Farzaneh N, Grassa CJ, McKown AD, Guy RD, Mansfield SD, Douglas CJ, Cronk QCB 2014 Landscape genomics of *Populus trichocarpa*: the role of hybridization, limited gene flow, and natural selection in shaping patterns of population structure. Evolution 68, 3260–3280. 25065449 10.1111/evo.12497

[CIT0047] Graham D, Patterson BD 1982 Responses of plants to low, nonfreezing temperatures: proteins, metabolism, and acclimation. Annual Review of Plant Physiology 33, 347–372.

[CIT0048] Grivet D, Sebastiani F, Alía R, Bataillon T, Torre S, Zabal-Aguirre M, Vendramin GG, González-Martínez SC 2011 Molecular footprints of local adaptation in two Mediterranean conifers. Molecular Biology and Evolution 28, 101–116. 20656795 10.1093/molbev/msq190

[CIT0049] Guerra D, Lamontanara A, Bagnaresi P, et al 2015 Transcriptome changes associated with cold acclimation in leaves of olive tree (*Olea europaea* L.). Tree Genetics & Genomes 11, 113.

[CIT0050] Gugger PF, Peñaloza-Ramírez JM, Wright JW, Sork VL 2017 Whole-transcriptome response to water stress in a California endemic oak, *Quercus lobata*. Tree Physiology 37, 632–644. 28008082 10.1093/treephys/tpw122

[CIT0051] Günther T, Coop G 2013 Robust identification of local adaptation from allele frequencies. Genetics 195, 205–220. 23821598 10.1534/genetics.113.152462 PMC3761302

[CIT0052] Hamel LP, Miles GP, Samuel MA, Ellis BE, Séguin A, Beaudoin N 2005 Activation of stress-responsive mitogen-activated protein kinase pathways in hybrid poplar (*Populus trichocarpa* × *Populus deltoides*). Tree Physiology 25, 277–288. 15631976 10.1093/treephys/25.3.277

[CIT0053] Hanin M, Brini F, Ebel C, Toda Y, Takeda S, Masmoudi K 2011 Plant dehydrins and stress tolerance: versatile proteins for complex mechanisms. Plant Signaling & Behavior 6, 1503–1509. 21897131 10.4161/psb.6.10.17088 PMC3256378

[CIT0054] Harfouche A, Meilan R, Altman A 2014 Molecular and physiological responses to abiotic stress in forest trees and their relevance to tree improvement. Tree Physiology 34, 1181–1198. 24695726 10.1093/treephys/tpu012

[CIT0055] Harrison LC, Weiser CJ, Burke MJ 1978 Environmental and seasonal factors affecting the frost-induced stage of cold acclimation in *Cornus stolonifera* Michx. Plant Physiology 62, 894–898. 16660633 10.1104/pp.62.6.894 PMC1092249

[CIT0056] Hasanuzzaman M, Nahar K, Alam MM, Roychowdhury R, Fujita M 2013 Physiological, biochemical, and molecular mechanisms of heat stress tolerance in plants. International Journal of Molecular Sciences 14, 9643–9684. 23644891 10.3390/ijms14059643 PMC3676804

[CIT0057] He F, Li H-G, Wang J-J, et al 2019 PeSTZ1, a C2H2-type zinc finger transcription factor from *Populus euphratica*, enhances freezing tolerance through modulation of ROS scavenging by directly regulating *PeAPX2*. Plant Biotechnology Journal 17, 2169–2183. 30977939 10.1111/pbi.13130 PMC6790368

[CIT0058] He F, Wang HL, Li HG, Su Y, Li S, Yang Y, Feng CH, Yin W, Xia X 2018 PeCHYR1, a ubiquitin E3 ligase from *Populus euphratica*, enhances drought tolerance via ABA-induced stomatal closure by ROS production in *Populus*. Plant Biotechnology Journal 16, 1514–1528. 29406575 10.1111/pbi.12893 PMC6041450

[CIT0059] Heer K, Ullrich KK, Hiss M, Liepelt S, Schulze Brüning R, Zhou J, Opgenoorth L, Rensing SA 2018 Detection of somatic epigenetic variation in Norway spruce via targeted bisulfite sequencing. Ecology and Evolution 8, 9672–9682. 30386566 10.1002/ece3.4374 PMC6202725

[CIT0060] Herman JJ, Sultan SE 2011 Adaptive transgenerational plasticity in plants: case studies, mechanisms, and implications for natural populations. Frontiers in Plant Science 2, 102. 22639624 10.3389/fpls.2011.00102 PMC3355592

[CIT0061] Hess M, Wildhagen H, Junker LV, Ensminger I 2016 Transcriptome responses to temperature, water availability and photoperiod are conserved among mature trees of two divergent Douglas-fir provenances from a coastal and an interior habitat. BMC Genomics 17, 682. 27565139 10.1186/s12864-016-3022-6 PMC5002200

[CIT0062] Hoban SM, Borkowski DS, Brosi SL, McCleary TS, Thompson LM, McLachlan JS, Pereira MA, Schlarbaum SE, Romero-Severson J 2016 Range-wide distribution of genetic diversity in the North American tree *Juglans cinerea*: a product of range shifts, not ecological marginality or recent population decline. Molecular Ecology 19, 4876–4891. 10.1111/j.1365-294X.2010.04834.x 21040046

[CIT0063] Hoffman DE, Jonsson P, Bylesjö M, Trygg J, Antti H, Eriksson ME, Moritz T 2010 Changes in diurnal patterns within the *Populus* transcriptome and metabolome in response to photoperiod variation. Plant, Cell & Environment 33, 1298–1313. 10.1111/j.1365-3040.2010.02148.x 20302601

[CIT0064] Hüve K, Bichele I, Tobias M, Niinemets Ü 2006 Heat sensitivity of photosynthetic electron transport varies during the day due to changes in sugars and osmotic potential. Plant, Cell & Environment 29, 212–228. 10.1111/j.1365-3040.2005.01414.x 17080637

[CIT0065] Ibáñez C, Kozarewa I, Johansson M, Ogren E, Rohde A, Eriksson ME 2010 Circadian clock components regulate entry and affect exit of seasonal dormancy as well as winter hardiness in *Populus* trees. Plant Physiology 153, 1823–1833. 20530613 10.1104/pp.110.158220 PMC2923903

[CIT0066] Jaskiewicz M, Conrath U, Peterhänsel C 2011 Chromatin modification acts as a memory for systemic acquired resistance in the plant stress response. EMBO Reports 12, 50–55. 21132017 10.1038/embor.2010.186 PMC3024125

[CIT0067] Jia J, Zhou J, Shi W, Cao X, Luo J, Polle A, Luo ZB 2017 Comparative transcriptomic analysis reveals the roles of overlapping heat-/drought-responsive genes in poplars exposed to high temperature and drought. Scientific Reports 7, 43215. 28233854 10.1038/srep43215 PMC5324098

[CIT0068] Johansson M, Ibáñez C, Takata N, Eriksson ME 2014 The perennial clock is an essential timer for seasonal growth events and cold hardiness. In: Staiger D, ed. Plant circadian networks: methods and protocols. Methods in molecular biology, vol. 1158. New York, NY: Springer New York, 297–311. 10.1007/978-1-4939-0700-7_20 24792060

[CIT0069] Joyce DG, Rehfeldt GE 2013 Climatic niche, ecological genetics, and impact of climate change on eastern white pine (*Pinus strobus* L.): guidelines for land managers. Forest Ecology and Management 295, 173–192.

[CIT0070] Kanehisa M, Goto S 2000 KEGG: Kyoto encyclopedia of genes and genomes. Nucleic Acids Research 28, 27–30. 10592173 10.1093/nar/28.1.27 PMC102409

[CIT0071] Karlberg A, Bako L, Bhalerao RP 2011 Short day-mediated cessation of growth requires the downregulation of AINTEGUMENTALIKE1 transcription factor in hybrid aspen. PLoS Genetics 7, e1002361. 22072988 10.1371/journal.pgen.1002361 PMC3207903

[CIT0072] Kim J-M, To TK, Ishida J, Matsui A, Kimura H, Seki M 2012 Transition of chromatin status during the process of recovery from drought stress in *Arabidopsis thaliana*. Plant & Cell Physiology 53, 847–856. 22505693 10.1093/pcp/pcs053

[CIT0073] Kozarewa I, Ibáñez C, Johansson M, Ögren E, Mozley D, Nylander E, Chono M, Moritz T, Eriksson ME 2010 Alteration of *PHYA* expression change circadian rhythms and timing of bud set in *Populus*. Plant Molecular Biology 73, 143–156. 20229130 10.1007/s11103-010-9619-2

[CIT0074] Krajmerová D, Hrivnák M, Ditmarová Ľ, Jamnická G, Kmeť J, Kurjak D, Gömöry D 2017 Nucleotide polymorphisms associated with climate, phenology and physiological traits in European beech (*Fagus sylvatica* L.). New Forests 48, 463–477.

[CIT0075] Kranner I, Minibayeva FV, Beckett RP, Seal CE 2010 What is stress? Concepts, definitions and applications in seed science. New Phytologist 188, 655–673. 20854396 10.1111/j.1469-8137.2010.03461.x

[CIT0076] Ksouri N, Jiménez S, Wells CE, Contreras-Moreira B, Gogorcena Y 2016 Transcriptional responses in root and leaf of *Prunus persica* under drought stress using RNA sequencing. Frontiers in Plant Science 7, 1715. 27933070 10.3389/fpls.2016.01715 PMC5120087

[CIT0077] Lafon-Placette C, Le Gac AL, Chauveau D, et al 2018 Changes in the epigenome and transcriptome of the poplar shoot apical meristem in response to water availability affect preferentially hormone pathways. Journal of Experimental Botany 69, 537–551. 29211860 10.1093/jxb/erx409

[CIT0078] Lämke J, Bäurle I 2017 Epigenetic and chromatin-based mechanisms in environmental stress adaptation and stress memory in plants. Genome Biology 18, 124. 28655328 10.1186/s13059-017-1263-6 PMC5488299

[CIT0079] Lane T, Best T, Zembower N, et al 2016 The green ash transcriptome and identification of genes responding to abiotic and biotic stresses. BMC Genomics 17, 702. 27589953 10.1186/s12864-016-3052-0 PMC5009568

[CIT0080] Lee E-J, Pei L, Srivastava G, et al 2011 Targeted bisulfite sequencing by solution hybrid selection and massively parallel sequencing. Nucleic Acids Research 39, e127–e127. 21785137 10.1093/nar/gkr598 PMC3201883

[CIT0081] Lei Y, Yin C, Li C 2006 Differences in some morphological, physiological, and biochemical responses to drought stress in two contrasting populations of *Populus przewalskii*. Physiologia Plantarum 127, 182–191.

[CIT0082] Lewontin RC, Krakauer J 1973 Distribution of gene frequency as a test of the theory of the selective neutrality of polymorphisms. Genetics 74, 175. 4711903 10.1093/genetics/74.1.175 PMC1212935

[CIT0083] Leyva-Pérez M de la O, Valverde-Corredor A, Valderrama R, Jiménez-Ruiz J, Muñoz-Merida A, Trelles O, Barroso JB, Mercado-Blanco J, Luque F 2015 Early and delayed long-term transcriptional changes and short-term transient responses during cold acclimation in olive leaves. DNA Research 22, 1–11. 25324298 10.1093/dnares/dsu033 PMC4379972

[CIT0084] Li B, Qin Y, Duan H, Yin W, Xia X 2011 Genome-wide characterization of new and drought stress responsive microRNAs in *Populus euphratica*. Journal of Experimental Botany 62, 3765–3779. 21511902 10.1093/jxb/err051 PMC3134338

[CIT0085] Li K-Q, Xu X-Y, Huang X-S 2016 Identification of differentially expressed genes related to dehydration resistance in a highly drought-tolerant pear, *Pyrus betulaefolia*, as through RNA-seq. PLoS ONE 11, e0149352. 26900681 10.1371/journal.pone.0149352 PMC4762547

[CIT0086] Li S, Lin Y-CJ, Wang P, et al 2019 The AREB1 transcription factor influences histone acetylation to regulate drought responses and tolerance in *Populus trichocarpa*. The Plant Cell 31, 663–686. 30538157 10.1105/tpc.18.00437 PMC6482633

[CIT0087] Li Y, Song Y, Xu B, Xie J, Zhang D, Cooke J 2017 Poplar CBF1 functions specifically in an integrated cold regulatory network. Tree Physiology 37, 98–115. 28175921 10.1093/treephys/tpw079

[CIT0088] Li Y, Zhao M, Motesharrei S, Mu Q, Kalnay E, Li S 2015 Local cooling and warming effects of forests based on satellite observations. Nature Communications 6, 6603. 10.1038/ncomms7603 PMC4389237 25824529

[CIT0089] Liang D, Zhang Z, Wu H, et al 2014 Single-base-resolution methylomes of *Populus trichocarpa* reveal the association between DNA methylation and drought stress. BMC Genetics 15, S9. 10.1186/1471-2156-15-S1-S9 PMC4118614 25080211

[CIT0090] Lichtenthaler HK 1996 Vegetation stress: an introduction to the stress concept in plants. Journal of Plant Physiology 148, 4–14.

[CIT0091] Lind-Riehl FJ, Gailing O 2017 Adaptive variation and introgression of a *CONSTANS*-like gene in North American red oaks. Forests 8, 3.

[CIT0092] Linhart YB, Grant MC 1996 Evolutionary significance of local genetic differentiation in plants. Annual Review of Ecology and Systematics 27, 237–277.

[CIT0093] Liu J, Feng L, Li J, He Z 2015 Genetic and epigenetic control of plant heat responses. Frontiers in Plant Science 6, 267. 25964789 10.3389/fpls.2015.00267 PMC4408840

[CIT0094] Liu S-C, Jin J-Q, Ma J-Q, Yao M-Z, Ma C-L, Li C-F, Ding Z-T, Chen L 2016 Transcriptomic analysis of tea plant responding to drought stress and recovery. PLoS ONE 11, e0147306. 26788738 10.1371/journal.pone.0147306 PMC4720391

[CIT0095] Liu Z, Jia Y, Ding Y, Shi Y, Li Z, Guo Y, Gong Z, Yang S 2017 Plasma membrane CRPK1-mediated phosphorylation of 14-3-3 proteins induces their nuclear import to fine-tune CBF signaling during cold response. Molecular Cell 66, 117–128.e5. 28344081 10.1016/j.molcel.2017.02.016

[CIT0096] Loarie SR, Duffy PB, Hamilton H, Asner GP, Field CB, Ackerly DD 2009 The velocity of climate change. Nature 462, 1052–1055. 20033047 10.1038/nature08649

[CIT0097] Lotterhos KE, Whitlock MC 2015 The relative power of genome scans to detect local adaptation depends on sampling design and statistical method. Molecular Ecology 24, 1031–1046. 25648189 10.1111/mec.13100

[CIT0098] Magalhães AP, Verde N, Reis F, Martins I, Costa D, Lino-Neto T, Castro PH, Tavares RM, Azevedo H 2016 RNA-seq and gene network analysis uncover activation of an ABA-dependent signalosome during the cork oak root response to drought. Frontiers in Plant Science 6, 1195. 26793200 10.3389/fpls.2015.01195 PMC4707443

[CIT0099] Magri D, Vendramin GG, Comps B, et al 2006 A new scenario for the Quaternary history of European beech populations: palaeobotanical evidence and genetic consequences. New Phytologist 171, 199–221. 16771995 10.1111/j.1469-8137.2006.01740.x

[CIT0100] Mai J, Herbette S, Vandame M, Cavaloc E, Julien J-L, Ameglio T, Roeckel-Drevet P, Oren R 2010 Contrasting strategies to cope with chilling stress among clones of a tropical tree, *Hevea brasiliensis*. Tree Physiology 30, 1391–1402. 20884609 10.1093/treephys/tpq075

[CIT0101] Mangul S, Martin LS, Langmead B, Sanchez-Galan JE, Toma I, Hormozdiari F, Pevzner P, Eskin E 2019 How bioinformatics and open data can boost basic science in countries and universities with limited resources. Nature Biotechnology 37, 324–326. 10.1038/s41587-019-0053-y 30833765

[CIT0102] Marron N, Dreyer E, Boudouresque E, Delay D, Petit JM, Delmotte FM, Brignolas F 2003 Impact of successive drought and re-watering cycles on growth and specific leaf area of two *Populus* × *canadensis* (Moench) clones, ‘Dorskamp’ and ‘Luisa_Avanzo’. Tree Physiology 23, 1225–1235. 14652222 10.1093/treephys/23.18.1225

[CIT0103] Martins K, Gugger PF, Llanderal-Mendoza J, González- Rodríguez A, Fitz-Gibbon ST, Zhao JL, Rodríguez-Correa H, Oyama K, Sork VL 2018 Landscape genomics provides evidence of climate-associated genetic variation in Mexican populations of *Quercus rugosa*. Evolutionary Applications 11, 1842–1858. 30459833 10.1111/eva.12684 PMC6231481

[CIT0104] McDowell N, Allen CD, Anderson-Teixeira K, et al 2018 Drivers and mechanisms of tree mortality in moist tropical forests. New Phytologist 219, 851–869. 29451313 10.1111/nph.15027

[CIT0105] Meireles JE, Beulke A, Borkowski DS, Romero-Severson J, Cavender-Bares J 2017 Balancing selection maintains diversity in a cold tolerance gene in broadly distributed live oaks. Genome 60, 762–769. 28683204 10.1139/gen-2016-0208

[CIT0106] Metzker ML 2010 Sequencing technologies — the next generation. Nature Reviews Genetics 11, 31–46. 10.1038/nrg2626 19997069

[CIT0107] Millar AJ, Urquiza U, Freeman PL, Hume A, Plotkin GD, Sorokina O, Zardilis A, Zielinski T 2019 Practical steps to digital organism models, from laboratory model species to ‘Crops *in silico*’. Journal of Experimental Botany 70, 2403–2418. 30615184 10.1093/jxb/ery435

[CIT0108] Mitchell-Olds T, Willis JH, Goldstein DB 2007 Which evolutionary processes influence natural genetic variation for phenotypic traits? Nature Reviews Genetics 8, 845–856. 10.1038/nrg2207 17943192

[CIT0109] Mittler R, Finka A, Goloubinoff P 2012 How do plants feel the heat? Trends in Biochemical Sciences 37, 118–125. 22236506 10.1016/j.tibs.2011.11.007

[CIT0110] Mittler R, Vanderauwera S, Gollery M, Van Breusegem F 2004 Reactive oxygen gene network of plants. Trends in Plant Science 9, 490–498. 15465684 10.1016/j.tplants.2004.08.009

[CIT0111] Modesto IS, Miguel C, Pina-Martins F, Glushkova M, Veloso M, Paulo OS, Batista D 2014 Identifying signatures of natural selection in cork oak (*Quercus suber* L.) genes through SNP analysis. Tree Genetics & Genomes 10, 1645–1660.

[CIT0112] Moran E, Lauder J, Musser C, Stathos A, Shu M 2017 The genetics of drought tolerance in conifers. New Phytologist 216, 1034–1048. 28895167 10.1111/nph.14774

[CIT0113] Morin X, Fahse L, Jactel H, Scherer-Lorenzen M, García-Valdés R, Bugmann H 2018 Long-term response of forest productivity to climate change is mostly driven by change in tree species composition. Scientific Reports 8, 1–12. 29618754 10.1038/s41598-018-23763-y PMC5884854

[CIT0114] Mosca E, Cruz F, Gómez-Garrido J, et al 2019 A reference genome sequence for the European silver fir (*Abies alba* Mill.): a community-generated genomic resource. G3 9, 2039–2049. 31217262 10.1534/g3.119.400083 PMC6643874

[CIT0115] Mosca E, Gugerli F, Eckert AJ, Neale DB 2016 Signatures of natural selection on *Pinus* cembra and *P. mugo* along elevational gradients in the Alps. Tree Genetics & Genomes 12, 9.

[CIT0116] Mousavi S, Alisoltani A, Shiran B, Fallahi H, Ebrahimie E, Imani A, Houshmand S 2014 *De novo* transcriptome assembly and comparative analysis of differentially expressed genes in *Prunus dulcis* Mill. in response to freezing stress. PLoS ONE 9, e104541. 25122458 10.1371/journal.pone.0104541 PMC4133227

[CIT0117] Mozgova I, Köhler C, Hennig L 2015 Keeping the gate closed: functions of the polycomb repressive complex PRC2 in development. The Plant Journal 83, 121–132. 25762111 10.1111/tpj.12828

[CIT0118] Müller M, Gailing O 2019 Abiotic genetic adaptation in the Fagaceae. Plant Biology 21, 783–795. 31081234 10.1111/plb.13008

[CIT0119] Mun B-G, Lee S-U, Park E-J, Kim H-H, Hussain A, Imran QM, Lee I-J, Yun B-W 2017 Analysis of transcription factors among differentially expressed genes induced by drought stress in *Populus davidiana*. 3 Biotech 7, 209. 10.1007/s13205-017-0858-7 PMC5493580 28667649

[CIT0120] Myburg A, Potts B, Marques CM, Kirst M, Gion J, Grattapaglia D, Grima-Pettenati J 2007 In: Kole CE, ed. Genome mapping and molecular breeding in plants. New York, NY: Springer, 115–160.

[CIT0121] Nadeau S, Meirmans PG, Aitken SN, Ritland K, Isabel N 2016 The challenge of separating signatures of local adaptation from those of isolation by distance and colonization history: the case of two white pines. Ecology and Evolution 6, 8649–8664. 28035257 10.1002/ece3.2550 PMC5192886

[CIT0122] Nakabayashi R, Saito K 2015 Integrated metabolomics for abiotic stress responses in plants. Current Opinion in Plant Biology 24, 10–16. 25618839 10.1016/j.pbi.2015.01.003

[CIT0123] Nystedt B, Street NR, Wetterbom A, et al 2013 The Norway spruce genome sequence and conifer genome evolution. Nature 497, 579–584. 23698360 10.1038/nature12211

[CIT0124] Pan Y, Birdsey RA, Fang J, et al 2011 A large and persistent carbon sink in the world’s forests. Science 333, 988–993. 21764754 10.1126/science.1201609

[CIT0125] Pan Y, Niu M, Liang J, Lin E, Tong Z, Zhang J 2017 Identification of heat-responsive miRNAs to reveal the miRNA-mediated regulatory network of heat stress response in *Betula luminifera*. Trees 31, 1635–1652.

[CIT0126] Papacek M, Christmann A, Grill E 2017 Interaction network of ABA receptors in grey poplar. The Plant Journal 92, 199–210. 28746755 10.1111/tpj.13646

[CIT0127] Pardo-Diaz C, Salazar C, Jiggins CD 2015 Towards the identification of the loci of adaptive evolution. Methods in Ecology and Evolution 6, 445–464. 25937885 10.1111/2041-210X.12324 PMC4409029

[CIT0128] Park Williams A, Allen CD, Macalady AK, et al 2013 Temperature as a potent driver of regional forest drought stress and tree mortality. Nature Climate Change 3, 292–297.

[CIT0129] Pashkovskiy PP, Vankova R, Zlobin IE, Dobrev P, Ivanov YV, Kartashov AV, Kuznetsov VV 2019 Comparative analysis of abscisic acid levels and expression of abscisic acid-related genes in Scots pine and Norway spruce seedlings under water deficit. Plant Physiology and Biochemistry 140, 105–112. 31091491 10.1016/j.plaphy.2019.04.037

[CIT0130] Paun O, Verhoeven KJF, Richards CL 2019 Opportunities and limitations of reduced representation bisulfite sequencing in plant ecological epigenomics. New Phytologist 221, 738–742. 30121954 10.1111/nph.15388 PMC6504643

[CIT0131] Peng S, Jiang H, Zhang S, Chen L, Li X, Korpelainen H, Li C 2012 Transcriptional profiling reveals sexual differences of the leaf transcriptomes in response to drought stress in *Populus yunnanensis*. Tree Physiology 32, 1541–1555. 23148036 10.1093/treephys/tps110

[CIT0132] Peng X, Wu Q, Teng L, Tang F, Pi Z, Shen S 2015 Transcriptional regulation of the paper mulberry under cold stress as revealed by a comprehensive analysis of transcription factors. BMC Plant Biology 15, 108. 25928853 10.1186/s12870-015-0489-2 PMC4432934

[CIT0133] Pina-Martins F, Baptista J, Pappas Jr G, Paulo OS 2019 New insights into adaptation and population structure of cork oak using genotyping by sequencing. Global Change Biology 25, 337–350. 30358018 10.1111/gcb.14497

[CIT0134] Rellstab C, Gugerli F, Eckert AJ, Hancock AM, Holderegger R 2015 A practical guide to environmental association analysis in landscape genomics. Molecular Ecology 24, 4348–4370. 26184487 10.1111/mec.13322

[CIT0135] Ren Y, Chen L, Zhang Y, Kang X, Zhang Z, Wang Y 2012 Identification of novel and conserved *Populus tomentosa* microRNA as components of a response to water stress. Functional & Integrative Genomics 12, 327–339. 22415631 10.1007/s10142-012-0271-6

[CIT0136] Reyna-López GE, Simpson J, Ruiz-Herrera J 1997 Differences in DNA methylation patterns are detectable during the dimorphic transition of fungi by amplification of restriction polymorphisms. Molecular and General Genetics 253, 703–710. 9079881 10.1007/s004380050374

[CIT0137] Richards CL, Alonso C, Becker C, et al 2017 Ecological plant epigenetics: evidence from model and non-model species, and the way forward. Ecology Letters 20, 1576–1590. 29027325 10.1111/ele.12858

[CIT0138] Roschanski AM, Csilléry K, Liepelt S, Oddou-Muratorio S, Ziegenhagen B, Huard F, Ullrich KK, Postolache D, Vendramin GG, Fady B 2016 Evidence of divergent selection for drought and cold tolerance at landscape and local scales in *Abies alba* Mill. in the French Mediterranean Alps. Molecular Ecology 25, 776–794. 26676992 10.1111/mec.13516

[CIT0139] Ruelland E, Zachowski A 2010 How plants sense temperature. Environmental and Experimental Botany 69, 225–232.

[CIT0140] Ruiz Daniels R, Taylor RS, Serra‐Varela MJ, Vendramin GG, González‐Martínez SC, Grivet D 2018 Inferring selection in instances of long‐range colonization: the Aleppo pine (*Pinus halepensis*) in the Mediterranean Basin. Molecular Ecology 27, 3331–3345. 10.1111/mec.14786 29972881

[CIT0141] Russell PH, Johnson RL, Ananthan S, Harnke B, Carlson NE 2018 A large-scale analysis of bioinformatics code on GitHub. PLoS ONE 13, e0205898. 30379882 10.1371/journal.pone.0205898 PMC6209220

[CIT0142] Ruttink T, Arend M, Morreel K, Storme V, Rombauts S, Fromm J, Bhalerao RP, Boerjan W, Rohde A 2007 A molecular timetable for apical bud formation and dormancy induction in poplar. The Plant Cell 19, 2370–2390. 17693531 10.1105/tpc.107.052811 PMC2002631

[CIT0143] Saidi Y, Finka A, Goloubinoff P 2011 Heat perception and signalling in plants: a tortuous path to thermotolerance. New Phytologist 190, 556–565. 21138439 10.1111/j.1469-8137.2010.03571.x

[CIT0144] Sakuma Y, Maruyama K, Osakabe Y, Qin F, Seki M, Shinozaki K, Yamaguchi-Shinozaki K 2006 Functional analysis of an *Arabidopsis* transcription factor, DREB2A, involved in drought-responsive gene expression. The Plant Cell 18, 1292–1309. 16617101 10.1105/tpc.105.035881 PMC1456870

[CIT0145] Sheffield J, Wood EF 2007 Characteristics of global and regional drought, 1950–2000: analysis of soil moisture data from off-line simulation of the terrestrial hydrologic cycle. Journal of Geophysical Research: Atmospheres 112, D17115.

[CIT0146] Shi Y, Ding Y, Yang S 2018 Molecular regulation of CBF signaling in cold acclimation. Trends in Plant Science 23, 623–637. 29735429 10.1016/j.tplants.2018.04.002

[CIT0147] Shuai P, Liang D, Zhang Z, Yin W, Xia X 2013 Identification of drought-responsive and novel *Populus trichocarpa* microRNAs by high-throughput sequencing and their targets using degradome analysis. BMC Genomics 14, 233. 23570526 10.1186/1471-2164-14-233 PMC3630063

[CIT0148] Sitch S, Huntingford C, Gedney N, et al 2008 Evaluation of the terrestrial carbon cycle, future plant geography and climate-carbon cycle feedbacks using five Dynamic Global Vegetation Models (DGVMs). Global Change Biology 14, 2015–2039.

[CIT0149] Sork VL, Fitz-Gibbon ST, Puiu D, Crepeau M, Gugger PF, Sherman R, Stevens K, Langley CH, Pellegrini M, Salzberg SL 2016 First draft assembly and annotation of the genome of a california endemic oak *Quercus lobata* Née (Fagaceae). G3 6, 3485–3495. 27621377 10.1534/g3.116.030411 PMC5100847

[CIT0150] Sow MD, Allona I, Ambroise C, et al 2018 Epigenetics in forest trees: state of the art and potential implications for breeding and management in a context of climate change. Advances in Botanical Research 88, 387–452.

[CIT0151] Srivastava R, Deng Y, Shah S, Rao AG, Howell SH 2013 BINDING PROTEIN is a master regulator of the endoplasmic reticulum stress sensor/transducer bZIP28 in *Arabidopsis*. The Plant Cell 25, 1416–1429. 23624714 10.1105/tpc.113.110684 PMC3663277

[CIT0152] Stark R, Grzelak M, Hadfield J 2019 RNA sequencing: the teenage years. Nature Reviews. Genetics 20, 631–656. 10.1038/s41576-019-0150-2 31341269

[CIT0153] Steane DA, Potts BM, McLean EH, Collins L, Holland BR, Prober SM, Stock WD, Vaillancourt RE, Byrne M 2017 Genomic scans across three Eucalypts suggest that adaptation to aridity is a genome-wide phenomenon. Genome Biology and Evolution 9, 253–265. 28391293 10.1093/gbe/evw290 PMC5381606

[CIT0154] Tang S, Dong Y, Liang D, Zhang Z, Ye C-Y, Shuai P, Han X, Zhao Y, Yin W, Xia X 2015 Analysis of the drought stress-responsive transcriptome of black cottonwood (*Populus trichocarpa*) using deep RNA sequencing. Plant Molecular Biology Reporter 33, 424–438.

[CIT0155] Tang W, Page M 2013 Transcription factor AtbZIP60 regulates expression of Ca^2+^-dependent protein kinase genes in transgenic cells. Molecular Biology Reports 40, 2723–2732. 23275191 10.1007/s11033-012-2362-9

[CIT0156] Teskey R, Wertin T, Bauweraerts I, Ameye M, McGuire MA, Steppe K 2015 Responses of tree species to heat waves and extreme heat events. Plant, Cell & Environment 38, 1699–1712. 10.1111/pce.12417 25065257

[CIT0157] Tian Q, Chen J, Wang D, Wang H-L, Liu C, Wang S, Xia X, Yin W 2017 Overexpression of a *Populus euphratica CBF4* gene in poplar confers tolerance to multiple stresses. Plant Cell, Tissue and Organ Culture 128, 391–407.

[CIT0158] Tiffin P, Ross-Ibarra J 2014 Advances and limits of using population genetics to understand local adaptation. Trends in Ecology & Evolution 29, 673–680. 25454508 10.1016/j.tree.2014.10.004

[CIT0159] Turchetto-Zolet AC, Pinheiro F, Salgueiro F, Palma-Silva C 2013 Phylogeographical patterns shed light on evolutionary process in South America. Molecular Ecology 22, 1193–1213. 23279129 10.1111/mec.12164

[CIT0160] Tuskan GA, Difazio S, Jansson S, et al 2006 The genome of black cottonwood, *Populus trichocarpa* (Torr. & Gray). Science 313, 1596–1604. 16973872 10.1126/science.1128691

[CIT0161] Vaseva I, Sabotič J, Sustar-vozlic J, Meglic V, Kidrič M, Demirevska K, Simova L 2012 The response of plants to drought stress: the role of dehydrins, chaperones, proteases and protease inhibitors in maintaining cellular protein function. In: Neves DF, Sanz JD, eds. Droughts: new research. Hauppauge, NY: Nova Science Publishers, 1–46.

[CIT0162] Vaultier MN, Cantrel C, Vergnolle C, Justin AM, Demandre C, Benhassaine-Kesri G, Ciçek D, Zachowski A, Ruelland E 2006 Desaturase mutants reveal that membrane rigidification acts as a cold perception mechanism upstream of the diacylglycerol kinase pathway in *Arabidopsis* cells. FEBS Letters 580, 4218–4223. 16839551 10.1016/j.febslet.2006.06.083

[CIT0163] Villar E, Klopp C, Noirot C, Novaes E, Kirst M, Plomion C, Gion JM 2011 RNA-seq reveals genotype-specific molecular responses to water deficit in eucalyptus. BMC Genomics 12, 538. 22047139 10.1186/1471-2164-12-538 PMC3248028

[CIT0164] Wang L, Su H, Han L, Wang C, Sun Y, Liu F 2014 Differential expression profiles of poplar MAP kinase kinases in response to abiotic stresses and plant hormones, and overexpression of PtMKK4 improves the drought tolerance of poplar. Gene 545, 141–148. 24780863 10.1016/j.gene.2014.04.058

[CIT0165] Wang Z, Gerstein M, Snyder M 2009 RNA-seq: a revolutionary tool for transcriptomics. Nature Reviews. Genetics 10, 57–63. 10.1038/nrg2484 PMC2949280 19015660

[CIT0166] Waring RH, Cleary BD 1967 Plant moisture stress: evaluation by pressure bomb. Science 155, 1248–1254. 17847540 10.1126/science.155.3767.1248

[CIT0167] Weiser CJ 1970 Cold resistance and injury in woody plants. Science 169, 1269–1278. 17772511 10.1126/science.169.3952.1269

[CIT0168] Weston DJ, Gunter LE, Rogers A, Wullschleger SD 2008 Connecting genes, coexpression modules, and molecular signatures to environmental stress phenotypes in plants. BMC Systems Biology 2, 16. 18248680 10.1186/1752-0509-2-16 PMC2277374

[CIT0169] Wren JD 2016 Bioinformatics programs are 31-fold over-represented among the highest impact scientific papers of the past two decades. Bioinformatics 32, 2686–2691. 27153671 10.1093/bioinformatics/btw284

[CIT0170] Xie Y, Chen P, Yan Y, et al 2018 An atypical R2R3 MYB transcription factor increases cold hardiness by CBF-dependent and CBF-independent pathways in apple. New Phytologist 218, 201–218. 29266327 10.1111/nph.14952

[CIT0171] Xing H, Fu X, Yang C, Tang X, Guo L, Li C, Xu C, Luo K 2018 Genome-wide investigation of pentatricopeptide repeat gene family in poplar and their expression analysis in response to biotic and abiotic stresses. Scientific Reports 8, 2817. 29434322 10.1038/s41598-018-21269-1 PMC5809412

[CIT0172] Xu J, Lu J, Evans R, Downes GM 2014 Relationship between ring width and tracheid characteristics in *Picea crassifolia*: implication in dendroclimatology. BioResources 9, 2203–2213.

[CIT0173] Xu J, Zhou S, Gong X, Song Y, van Nocker S, Ma F, Guan Q 2018 Single-base methylome analysis reveals dynamic epigenomic differences associated with water deficit in apple. Plant Biotechnology Journal 16, 672–687. 28796917 10.1111/pbi.12820 PMC5787839

[CIT0174] Yakovlev I, Fossdal CG, Skrøppa T, Olsen JE, Jahren AH, Johnsen Ø 2012 An adaptive epigenetic memory in conifers with important implications for seed production. Seed Science Research 22, 63–76.

[CIT0175] Ye G, Ma Y, Feng Z, Zhang X 2018 Transcriptomic analysis of drought stress responses of sea buckthorn (*Hippophae rhamnoides* subsp. *sinensis*) by RNA-seq. PLoS ONE 13, e0202213. 30102736 10.1371/journal.pone.0202213 PMC6089444

[CIT0176] Yu D, Wildhagen H, Tylewicz S, Miskolczi PC, Bhalerao RP, Polle A 2019 Abscisic acid signalling mediates biomass trade-off and allocation in poplar. New Phytologist 223, 1192–1203. 31050802 10.1111/nph.15878

[CIT0177] Yu J, Ge H, Wang X, Tang R, Wang Y, Zhao F, Lan W, Luan S, Yang L 2017 Overexpression of pyrabactin resistance-like abscisic acid receptors enhances drought, osmotic, and cold tolerance in transgenic poplars. Frontiers in Plant Science 8, 1752. 29081783 10.3389/fpls.2017.01752 PMC5645508

[CIT0178] Yu Z, Wang X, Zhang L 2018 Structural and functional dynamics of dehydrins: a plant protector protein under abiotic stress. International Journal of Molecular Sciences 19, E3420. 30384475 10.3390/ijms19113420 PMC6275027

[CIT0179] Zardilis A, Hume A, Millar AJ 2019 A multi-model framework for the *Arabidopsis* life cycle. Journal of Experimental Botany 70, 2463–2477. 31091320 10.1093/jxb/ery394 PMC6487595

[CIT0180] Zhang J, Li J, Liu B, Zhang L, Chen J, Lu M 2013 Genome-wide analysis of the *Populus Hsp90* gene family reveals differential expression patterns, localization, and heat stress responses. BMC Genomics 14, 532–546. 23915275 10.1186/1471-2164-14-532 PMC3750472

[CIT0181] Zhang J, Liu B, Li J, Zhang L, Wang Y, Zheng H, Lu M, Chen J 2015 *Hsf* and *Hsp* gene families in *Populus*: genome-wide identification, organization and correlated expression during development and in stress responses. BMC Genomics 16, 181. 25887520 10.1186/s12864-015-1398-3 PMC4373061

[CIT0182] Zhang Q, Cai M, Yu X, Wang L, Guo C, Ming R, Zhang J 2017 *a* Transcriptome dynamics of *Camellia sinensis* in response to continuous salinity and drought stress. Tree Genetics & Genomes 13, 78.

[CIT0183] Zhang S, Zhang L, Zhao Z, Li Y, Zhou K, Su L, Zhou Q 2016 Root transcriptome sequencing and differentially expressed drought-responsive genes in the *Platycladus orientalis* (L.). Tree Genetics & Genomes 12, 79.

[CIT0184] Zhang X, Teixeira da Silva JA, Niu M, Li M, He C, Zhao J, Zeng S, Duan J, Ma G 2017 *b* Physiological and transcriptomic analyses reveal a response mechanism to cold stress in *Santalum album* L. leaves. Scientific Reports 7, 42165. 28169358 10.1038/srep42165 PMC5294638

[CIT0185] Zhou Z, Ma H, Lin K, Zhao Y, Chen Y, Xiong Z, Wang L, Tian B 2015 RNA-seq reveals complicated transcriptomic responses to drought stress in a nonmodel tropic plant, *Bombax ceiba* L. Evolutionary Bioinformatics11s1, doi:10.4137/EBO.S20620. 10.4137/EBO.S20620 PMC4479181 26157330

