# Supplementary material for: Molecular bases of responses to abiotic stress in trees
Source: J Exp Bot. 2019 Nov 26;71(13):3765–79. doi: 10.1093/jxb/erz532 (PMC7316969; doi:10.1093/jxb/erz532)
Supplement: erz532_suppl_Supplementary_Table_S1-S2 [file erz532_suppl_supplementary_table_s1-s2.pdf]

Supplementary Table 1. Bioinformatic tools used in RNA-seq studies of tree species

Analysis	Tools	References
**Assembly**	Trinity (*de novo*)	3-5, 12, 15, 17, 19, 22-26
	TopHat/Cufflinks (with reference)	2, 11, 18
	AbySS (*de novo*)	13
	Newbler (*de novo*)	16
**Mapping**	Bowtie/Bowtie2	3, 7-9, 13, 17, 23-25
	SOAP2	4, 14, 20, 21
	BWA	6, 16, 26
	STAR	2
	CLC Genomics Workbench	5
	TopHat	10
**Quantification**	RSEM	3, 15, 17, 22, 24-26
	ERANGE	4, 14, 19, 20
	samtools	6-8
	Cufflinks/Cuffdiff	10, 11, 18
	CLC Genomics Workbench	5
	HTSeq	12
	DNAstar Qseq	13
	eXpress	23
**Differential expression**	DESeq/DESeq2	1, 5, 6, 12, 16, 22, 24
	DEGseq	1, 7, 8, 10
	Audic and Claverie (1997) test	4, 14, 20
	NOIseq	1, 25
	edgeR	3, 23
	Cuffdiff	11, 18
	EBSeq	15, 17
	DNAstar Qseq	13
	IDEG6	19
	GFOLD	26
**GO enrichment**	agriGO	6, 10, 17, 20
	WEGO	4, 14, 16
	BLAST2GO	3, 16
	BiNGO	12, 15
	topGO	9
	GOSSIP	13
	GOseq	22
	GOATOOLS	24

NOTE:

References are as follows (see Main Text bibliography): **1**: Behringer *et al.*, 2015. **2**: Filichkin *et al.*, 2018. **3**: Fox *et al.*, 2017. **4**: Gao *et al.*, 2015. **5**: Guerra *et al.*, 2015. **6**: Gugger *et al.*, 2016. **7**: Haider *et al.*, 2017a. **8**: Haider *et al.*, 2017b. **9**: Hess *et al.*, 2016. **10**: Jia *et al.*, 2017. **11**: Ksouori *et al.*, 2016. **12**: Lane *et al.*, 2016. **13**: Leyva-Pérez *et al.*, 2014. **14**: Li *et al.*, 2016. **15**: Liu *et al.*, 2016. **16**: Magalhaes *et al.*, 2016. **17**: Mousavi *et al.*, 2014. **18**: Mun *et al.*, 2017. **19**: Peng *et al.*, 2015. **20**: Tang *et al.*, 2015. **21**: Xing *et al.*, 2018. **22**: Ye *et al.*, 2018. **23**: Zhang Q *et al.*, 2017. **24**: Zhang S *et al.*, 2016. **25**: Zhang X *et al.*, 2017. **26**: Zhou *et al.*, 2015.

Supplementary Table 2. Description and functional annotation of candidate genes (and their SNPs) in response to drought stress and temperature showing departure from neutrality along environmental gradients.

Species	Functional category	Description	Trait	Genetic Marker	Outlier test	GEA[a]	PS[b]	Ref[c]
Fagus sylvatica	Electron transport	Ubiquinol-cytochrome c reductase iron-sulfur subunit	Abiotic stress	SNPs	Arlequin, BayeScan	-	-	Csilléry et al. 2014
	Photosynthesis	Photosystem II light harvesting complex protein						
	-	Continuous vascular ring						
	Oxidoreductase	Aldehyde Dehydrogenase (ALDH)	Environmental adaptation	SNPs	Arlequín, BayeScan, LOSITAN	Bayenv2, Samβada	Yes (nuSSRs)	Cuervo-Alarcon et al. 2018
	Oxidoreductase	Ascorbate peroxidase (APX)						
	Response to drought stress	Early responsive to dehydration (ERD)						
	Hidrolase	Cysteine proteinase (CysPro)						
	Oxidoreductase	Isocitrate dehydrogenase (IDH), NADP dependent						
	Transcription factor	Diacylglycerol (DAG)						
	Transcription factor	NAC transcription factor (NAC)						
	Transferase	Xyloglucan endotransglucosylase/hydrolase (XTH)						
	Oxidoreductase	Short-chain dehydrogenases/reductase (SDR)						
	Transporter	Potassium transport 2 (KT2)						
	Transcription factor	Dehydration responsive element-binding protein (DREB)						
	Hidrolase	Adenosylhomocysteinase (SAHH)						

	Hydrolase, Protein phosphatase	Protein phosphatase 2C (PP2C)						
	Oxidoreductase<br>Oxidoreductase<br>Ligase<br>Hydrolase, Protein phosphatase	Superoxide dismutase (SOD1)<br>Aldehyde dehydrogenase (ALDH)<br>Glutamine amidotransferase (GAT) or asparagine synthase<br>Protein phosphatase 2C (PP2C)	Drought	Gene expression (q-PCR)	-	Principal components analysis (PCA)	Yes (nuSSRs)	Carsjens et al. 2014
	Drought stress (LEA gene family)<br>DNA binding	Dehydrin<br>Histone 3	Drought<br>Temperature and Precipitation	SNPs	BayeScan, LOSITAN	Samβada, TASSEL	Yes (nuSSRs)	Krajmerová et al. 2017
Quercus suber	Ligase<br>Chaperone<br>Transcription factor	Glutamine synthetase nodule isozyme (GS)<br>Class I small heat shock protein (sHSP)<br>Auxin response factor 16 (ARF16)	Temperature<br>Drought	SNPs	ARLEQUIN	-	Yes (Three potential scenarios of genetic structure were tested)	Modesto et al. 2014
	Metabolism<br>Oxidoreductase, Peroxidase<br>Binding, Transcription Regulator<br>Binding, Transcription Regulator	Trehalase (TRE1)<br>Peroxidase 47 (PER47)<br>Cleavage and polyadenylation specificity factor subunit 1 (CPSF160)<br>Activating signal cointegrator 1 complex subunit 1 (Ascc1)	Temperature and Precipitation	SNPs	SELESTIM, BayeScan	Baypass	Yes (from SNPs: PCA and MAVERICK programs)	Pina Martins et al. 2019

	Transferase	Beta-glucuronosyltransferase (GlcAT14A)						
	GTP binding	Guanylate-binding protein 6 (GBP6)						
	DNA-binding transcription factor activity	Protein CUPSHAPED COTYLEDON 2 (NAC098)						
	Endopeptidase activity	Thylakoidal processing peptidase 1 (TPP1)						
	Binding, Transcription Regulator	Pentatricopeptide repeat-containing protein						
Quercus rubra and *Q. ellipsoidallis*	Transcription factors	Constant-like (COL)	Drought	EST-SSRs	LOSITAN	-	Yes (nuSRR)	Lind-Reihl et al. 2014
Quercus petrea, Q. pubescens and *Q. robur*	Transferase	Galactinol synthase 1 (GolS1)	Precipitation	SNPs	-	Latent Factor Mixed Models-LFMMs	Yes	Rellstab et al. 2016
	Regulation of circadian rhythm and photoperiodic flowering	Gigantea (G1) protein						
Quercus lobata	-	Auxin-repressed protein (AUX-REP)	Budburst	SNPs	Hierarchical F statistics	Vegan package in R	Yes (SSRs)	Sork et al. 2016
	Endopeptidase inhibitor activity (Hydrolase)	Alpha-amylase/subtilisin inhibitor						
	Chaperone	class I small heat shock protein (HSP17.4)	Temperature					
	Binding	Translation elongation factor-1, alpha-subunit (EF1A)						
	Cold acclimation	Inducer of CBF expression (ICE1)	Freezing	coding seq; SNPs	BayeScan	GLM (R environment)	Yes (SSRs, cpDNA)	Meireles et al. 2017

Quercus virginiana, Q. oleoides, Q. geminata, Q. minima, Q. fusiformis, Q. sangreana,, Q. brandegeei	Cold acclimation	High expression of osmotically responsive (HOS1)						
	Oxidoreductase, Transferase	Glyceraldehyde-3-phosphate dehydrogenase subunit B (GAPDB)						
	Chromatin regulator	Chromatin remodeling protein (CHR11)						
Eucalyptus camaldulensis	Transfarase	Caffeate 3-O-methyltransferase 1 (COMT)	Drought	SNP	Fdist, BayeScan	MatSAM, TASSEL	Yes (Simulated data set based on 15 putatively nuSSRs)	Dillon et al. 2014
	Drought stress (LEA gene family)	Dehydrin like protein						
	Receptor	Erecta leucine rich repeat protein (ERECTA)						
	Aquaporin	Plasma membrane intrinsic protein (PIP2)						
Eucalyptus gamaphocephala	Binding	CONSTANS-like protein CO1	Precipitation, aridity	EST-SSRs	BayeScan	TASSEL	Yes (SSR)	Bradbury et al. 2013
	Oxidoreductase	Quinone oxidoreductase						
			Temperature					

Eucalytustricarpa, E. salubris, E. loxopleba	Transferase<br>Protease<br>-	ATP phosphoribosyltransferase<br>Subtilisin like protease<br>Disease resistance protein	Precipitation, Temperature, Aridity	DArTseq	BayeScan	Linear Regression (PROC REG procedure by SAS software)	-	Steane et al. 2016
Populus trichocarpa	-	Rab GTPase homolog A4a	Temperature, precipitation	SNP array	Fdist, BayeScan	Bayenv2	Yes (Admixture version 1.22 and PCA)	Geraldes et al. 2014
	Tanscriptional repressors	Pseudo-response regulator 5(PRR5)						
	Molecular adaptor activity	ARM repeat superfamily protein						
	Transcription factor	AP2 domain-containing transcription factor						
	Transcription repressor	Leunig_ homolog (LUH)						
	Transferase	Phosphatidate cytidylyltransferase family protein						
	Transporter	Nitrate transporter 2:1 (NRT2:1)						
	DNA-Binding	Homeobox-leucine zipper protein REVOLUTA (REV)						
	Aquaporin	Plasma membrane protein 3 (PIP3)						
	Transferase	O-Fucosyltransferase family protein						
	DNA binding, Transcription regulator	Transparent testa glabra 1 (TTG1)						
	Oxidoreductase	Laccase 3 (LAC3)						
	DNA binding	AHBP-1B; DNA binding						
	Proton antiporter activity	Salt overly sensitive (SOS1)						
	Transporter	ABC transporter						

Pinus strobus	Transcription factor	Basic helix- loop- helix (bHLH) DNA- binding superfamily protein	Water deprivation	SNPs	-	Bayenv2, LFMM	Yes (SSRs)	Nadeau et al. 2016
	Regulation of chloroplast-actin (cp-actin) filaments	Plastid movement impaired1-related1 (PMIR1)						
	Binding	Serine–threonine- protein kinase						
	Binding	Serine–threonine- protein kinase						
	Transferase	Galacturonosyltransferase 13-like	Phenology, growth					
Pinus monticola	Transport membrane	TOM1- like protein 2	Phenology, growth					
Pinus taeda	-	Hypothetical proteins; Ca+2 dependent kinase;K+:H+ antiporter; TIFY domain-containing protein; BAG protein; Dehydratase like protein; Thioredoxin-related protein; PTAC2 like protein; LIM transcription factor; PPR protein; Histone 2A protein	Temperature and precipitation as drivers of distribution	SNPs	GENETICS, HIERFSTAT	Statistical correlations and Bayes factor	Yes (SSRs, isozymes)	Eckert et al. 2010a
	Transporter	Hexose:hydrogen symporter	Aridity	SNPs and SSRs	Fdist2	Statistical correlations; PCA	Yes (SSRs)	Eckert et al. 2010b
	Oxidoreductase	Photosystem II protein						
	Metal-binding	C3HC4-type RING finger						
	Transporter	MATE efflux family protein						
	Transporter	UDP-galactose transporter						
	Terpenoid synthesis	Myrcene synthase, malate synthase, cytochrome P450, shikimate O-hydroxycinnamoyltransferase,	Temperature, precipitation	SNPs (exome capture)	OUtFlank, spatial ancestry	TASSEL; redundancy analysis; Samβada	-	Lu et al. 2019

		(−)-alpha-pinene synthase, (−)-alpha-terpineol synthase			analysis (SPA)			
	Transcription factor	bHLH, MADS, MYB, GRAS						
	Abiotic stress response	Asparagine synthetase, 2-oxoisovalerate dehydrogenase, late embryogenesis abundant protein, WAT1-related protein, bark storage protein A-like						
Pinus pinaster	Cell wall formation	Putative Arabinogalactan/proline-rich protein (PR-AGP4)	Drought stress	CG	BayesFst, Fdist2	-	Yes (SSRs)	Eveno et al. 2008
	Cell wall formation	Put. Arabinogalactan/glycin-rich protein (GRP3)						
	Putative methyltransferase with SAM-binding domain	Early response to dehydration (erd3)						
	Drought stress (LEA gene family)	Dehydrins (dhn1, dhn2)						
	Drought stress (belong to the ASR [abscisic-,stress-, and ripening-induced] gene family)	lp3-l						
Pinus cembra	Response to stress	60S acidic ribosomal protein P0 (RPP0A)	Temperature	SNPs	-	Bayenv, LFMM	Yes (previous study)	Mosca et al. 2016
P. cembra and P.	Chaperone	Heat shock protein 101 (Hsp)						
	Oxidoreductase, Peroxidase	Respiratory burst oidase homologue D (RBOHD)						

	Transferase	Cellulose synthase/transferase (CESA UDP-forming)						
Pinus mugo	Transporter	Calcium-sodium antiporter (CAX11)						
	Transferase	Glutathione transferasa protein (GST)						
	Raffinose catabolism	Raffinose-specific alpha-galactosidase						
Abies alba	Chaperone	Heat shock protein 83-like (HSP)	Cold Temperature, drought	SNPs	Outlier detection: Fdist, FLK, Bayescan	LFMM	Yes	Roschanski et al. 2016
	Transferase	Mannose-1-phosphate guanylyltransferase 1-like (CYT1)						
	Oxidoreductase	Succinate dehydrogenase (SDH)						
	Peptidase	ATP-dependent Clp protease proteolytic subunitrelated protein chloroplastic-like (clpP1)						
	Binding	Actin-like isoform x1						
	Structural constituent of ribosome	60S ribosomal protein l7-4-like isoform x2						
	Ligase	Magnesium chelatase subunit chloroplastic-like (CHLI1)						
	Allosteric enzyme, Oxidoreductase	Ribonucleoside diphosphate reductase large subunit						
Pinus halepensis	Nucleotide binding-stress response	ABC transporter G family member 11-like (ABCG11)	Droguht, aridity	SNPs	PCADAPT	Bayenv2	Yes (SSRs)	Ruiz Daniels et al. 2017
	Identical protein binding	Peroxisomal membrane protein 11D-like						
	Transferase	RING-H2 finger protein ATL48-like (ATL48)						

Pinus strobus	Transporter	Multidrug resistance associated protein 1 (MRP)	Cold and drought stresses, photoperiodic response, phenology, growth, development	SNPs, SSRs	Bayescan	Spearman rank correlations, Redundancy Analysis	Yes (SSRs SNPs)	Rajora et al. 2016
	Transferase	Glutathione S-transferase (GST)						

[a] GEA: genotype-environment association

[b] PS: Population Structure Test

[c] Ref: References

References:

Bradbury D, Smithson A, Krauss SL. 2013. Signatures of diversifying selection at EST-SSR loci and association with climate in natural Eucalyptus populations. Molecular Ecology 22, 5112-5129.

Carsjens C, Nguyen Ngoc Q, Guzy J, Knutzen F, Meier IC, Müller M, Finkeldey R, Leuschner C, Polle A. 2014. Intra-specific variations in expression of stress-related genes in beech progenies are stronger than drought-induced responses. Tree Physiology 34 (12), 1348-1361.

Csilléry K, Lalagüe H, Vendramin GG, González-Martínez SC, Fady B and Oddou-Muratorio S. 2014. Detecting short spatial scale local adaptation and epistatic selection in climate-related candidate genes in European beech (*Fagus sylvatica*) populations. Molecular Ecology 23, 4696-4708.

Cuervo-Alarcon L, Arend M, Müller M, Sperisen C, Finkeldey R, Krutovsky KV. 2018. Genetic variation and signatures of natural selection in populations of European beech (*Fagus sylvatica* L.) along precipitation gradients. Tree Genetics & Genomes 14 (84), 1-21

Dillon S, McEvoy R, Baldwin DS, Rees GN, Parsons Y, Southerton S. 2014. Characterisation of adaptive genetic diversity in environmentally contrasted populations of *Eucalyptus camaldulensis* Dehnh. (river red gum). PLOS ONE 9 (8), e103515. https://doi.org/10.1371/journal.pone.0103515

Eckert CG, Samis KE and Lougheed SC 2010a. Genetic variation across species' geographical ranges: the central-marginal hypothesis and beyond Molecular Ecology 17: 1170-1188

Eckert AJ, van Heerwaarden J, Wegrzyn JL, Nelson CD, Ross-Ibarra J, González-Martínez SC and Neale DB. 2010b. Patterns of population structure and environmental associations to aridity across the range of loblolly Pine (*Pinus taeda* L., Pinaceae). Genetics 185: 969-982.

Eveno E, Collada C, Guevara MA, Léger V, Soto A, Díaz L, Léger P, González-Martínez SC, Cervera MT, Plomion C and Garnier-Gére PH. 2008. Contrasting patterns of selection at *Pinus pinaster* Ait. drought stress candidate genes as revealed by genetic differentiation analyses. Molecular Biology and Evolution 25:417–437.

Geraldes A, Farzaneh N, Grassa CJ, McKown AD, Guy RD, Mansfield SD, Douglas SJ, Cronk QCB. 2014. Landscape genomics of *Populus trichocarpa*: the role of hybridization, limited gene flow, and natural selection in shaping patterns of population structure. Evolution 68 (11): 3260-3280.

Krajmerová D, Hrivnák M, Ditmarová Ľ, Jamnická G, Kmeť J, Kurjak D, Gömöry D. 2017. Nucleotide polymorphisms associated with climate, phenology and physiological traits in European beech (*Fagus sylvatica* L.). New Forests 48: 463.

Lind-Riehl JF, Sullivan AR, Gailing O. 2014. Evidence for selection on a CONSTANS-like gene between two red oak species. Annals of Botany 113 (6), 967–975

Lu M, Loopstra CA and Krutovsky KV 2019. Detecting the genetic basis of local adaptation in loblolly pine (*Pinus taeda* L.) using whole exome-wide genotyping and an integrative landscape genomics analysis approach. Ecology and Evolution 9: 6798-6809

Meireles JE, Beulke A, Borkowski DS, Romero-Severson J, Cavender-Bares J. 2017. Balancing selection maintains diversity in a cold tolerance gene in broadly distributed live oaks. Genome 60 (9),762-69.

Modesto IS, Miguel C, Pina-Martins F, Glushkova M, Veloso M, S. Paulo O, Batista D. 2014. Identifying signatures of natural selection in cork oak (*Quercus suber* L.) genes through SNP analysis. Tree Genetics & Genomes 10 (6), 1645-1660.

Mosca E, Gugerly F, Eckert AJ and Neale DB. 2016. Signatures of natural selection on *Pinus cembra* and *P. mugo* along elevational gradients in the Alps. Tree Genetics and Genomes 12: 9. https://doi.org/10.1007/s11295-015-0964-9

Nadeau S, Meirmans PG, Aitken SN, Ritland K, Isabel N. 2016. The challenge of separating signatures of local adaptation from those of isolation by distance and colonization history: The case of two white pines. Ecology and Evolution 6 (24), 8649-8664.

Pina-Martins F, Baptista J, Pappas Jr G, Paulo OS. 2019. New insights into adaptation and population structure of cork oak using genotyping by sequencing. Global Change Biology 25 (1), 337-350.

Rellstab C, Zoller S, Walthert L, Lesur I, Pluess AR, Graf R, Bodénès C, Sperisen C, Kremer A and Gugerli F. 2016. Signatures of local adaptation in candidate genes of oaks (*Quercus* spp.) with respect to present and future climatic conditions. Molecular Ecology 25, 5907-5924.

Sork VL, Squire K, Gugger PF, Steele SE, Levy ED, Eckert AJ. 2016. Landscape genomic analysis of candidate genes for climate adaptation in a California endemic oak, *Quercus lobata*. American Journal of Botany 103 (1), 33-46.

Steane DA, Potts BM, McLean EH, Collins L, Holland BR, Prober SM, Stock WD, Vaillancourt RE, Byrne M. 2017. Genomic Scans across three Eucalypts suggest that adaptation to aridity is a genome-wide phenomenon. Genome Biology and Evolution 9 (2), 253-265.

Rajora OP, Eckert AJ and Zinck JWR 2016. Single-locus versus multilocus patterns of local adaptation to climate in eastern white Pine (*Pinus strobus*, Pinaceae). PLOS One 11(7): e0158691. doi:10.1371/journal.pone.0158691

Roschanski AM, Csilléry K, Liepelt S, Oddou-Muratorio S, Ziegenhagen B, Huard F, Ullrich KK, Postolache D, Vendramin GG, Fady B. Evidence of divergent selection for drought and cold tolerance at landscape and local scales in *Abies alba* Mill. in the French Mediterranean Alps. Molecular Ecology 25 (3), 776-94.

Ruiz Daniels R,Taylor RS, Serra-Varela MJ, Vendramin GG, González-Martínez SC and Grivet D. 2018. Inferring selection in instances of long-range colonization: The Aleppo pine (*Pinus halepensis*) in the Mediterranean Basin. Molecular Ecology 27:3331–3345.
